# Bringing Safe and Standardized Cell Therapies to Industrialized Processing for Burns and Wounds

**DOI:** 10.3389/fbioe.2020.00581

**Published:** 2020-06-19

**Authors:** Alexis Laurent, Poyin Lin, Corinne Scaletta, Nathalie Hirt-Burri, Murielle Michetti, Anthony S. de Buys Roessingh, Wassim Raffoul, Bin-Ru She, Lee Ann Applegate

**Affiliations:** ^1^Tec-Pharma SA, Bercher, Switzerland; ^2^Regenerative Therapy Unit, Lausanne University Hospital, University of Lausanne, Epalinges, Switzerland; ^3^Transwell Biotech Co. Ltd., Hsinchu, Taiwan; ^4^Pediatric Surgery Service, Lausanne University Hospital, University of Lausanne, Lausanne, Switzerland; ^5^Plastic, Reconstructive & Hand Surgery Service, Lausanne University Hospital, University of Lausanne, Lausanne, Switzerland; ^6^Oxford Suzhou Center for Advanced Research, Science and Technology Co. Ltd., Oxford University, Suzhou, China; ^7^Competence Center for Applied Biotechnology and Molecular Medicine, University of Zurich, Zurich, Switzerland

**Keywords:** cell therapy, clinical cell banking, progenitor cells, fibroblasts, GMP manufacturing, burns, chronic wounds

## Abstract

Cultured primary progenitor cell types are worthy therapeutic candidates for regenerative medicine. Clinical translation, industrial transposition, and commercial implementation of products based on such cell sources are mainly hindered by economic or technical barriers and stringent regulatory requirements. Applied research in allogenic cellular therapies in the Lausanne University Hospital focuses on cell source selection technique optimization. Use of fetal progenitor cell sources in Switzerland is regulated through Federal Transplantation Programs and associated Fetal Biobanks. Clinical applications of cultured primary progenitor dermal fibroblasts have been optimized since the 1990s as “Progenitor Biological Bandages” for pediatric burn patients and adults presenting chronic wounds. A single organ donation procured in 2009 enabled the establishment of a standardized cell source for clinical and industrial developments to date. Non-enzymatically isolated primary dermal progenitor fibroblasts (FE002-SK2 cell type) served for the establishment of a clinical-grade Parental Cell Bank, based on a patented method. Optimized bioprocessing methodology for the FE002-SK2 cell type has demonstrated that extensive and consistent progenitor cell banks can be established. *In vitro* mechanistic characterization and *in vivo* preclinical studies have confirmed potency, preliminary safety and efficacy of therapeutic progenitor cells. Most importantly, highly successful industrial transposition and up-scaling of biobanking enabled the establishment of tiered Master and Working Cell Banks using Good Manufacturing Practices. Successive and successful transfers of technology, know-how and materials to different countries around the world have been performed. Extensive developments based on the FE002-SK2 cell source have led to clinical trials for burns and wound dressing. Said trials were approved in Japan, Taiwan, USA and are continuing in Switzerland. The Swiss Fetal Transplantation Program and pioneer clinical experience in the Lausanne Burn Center over three decades constitute concrete indicators that primary progenitor dermal fibroblasts should be considered as therapeutic flagships in the domain of wound healing and for regenerative medicine in general. Indeed, one single organ donation potentially enables millions of patients to benefit from high-quality, safe and effective regenerative therapies. This work presents a technical and translational overview of the described progenitor cell technology harnessed in Switzerland as cellular therapies for treatment of burns and wounds around the globe.

## Introduction

Therapeutic developments driving regenerative medicine increasingly democratize cell-based therapies for treatment and prevention of wide arrays of acute and degenerative afflictions. Demographic shifts promoting higher incidence of chronic disease have prompted swift expansion of translational work pertaining to skin wound care, in particular to repair and restore function or supplement traditional therapeutic management (Vacanti and Langer, 1999; Marks and Gottlieb, 2018). The main indications of topical cell-based therapies remain set on complex, poorly-healing cutaneous affections. Corresponding clinical cases comprise ulcers, deep partial-thickness burn wounds or donor site wounds, which are highly demanding in classical wound care or necessitate skin grafting (Hernon et al., 2006; Li and Maitz, 2018). In such a context, wherein patient and practitioner expectations fall second highest only to regulatory requirements, cultured primary progenitor cells and derivatives have been demonstrably identified as worthy therapeutic candidates (Hebda and Dohar, 1999; Metcalfe and Ferguson, 2008; Larijani et al., 2015). Clinical-grade GMP-validated (Good Manufacturing Practices) allogenic progenitor cell sources trigger utmost interest. Indeed, pragmatic wielding of their tremendous therapeutic potential can minimize delays in medicinal product availability for the patient and provide maximal homogeneity, safety and stability of both biological starting materials and end-products (De Buys Roessingh et al., 2006; Applegate et al., 2009).

Multi- and inter-disciplinary professional approaches are instrumental in achieving implementation through clinical translation of allogenic cell-based therapies (Marks and Gottlieb, 2018). Market-approvals of Advanced Therapy Medicinal Products (ATMPs), although formidably tedious and complex to acquire, fall short of the challenge consisting in harnessing the optimal cell source (Applegate et al., 2009; Marks and Gottlieb, 2018). The latter must meet high quality standards, related to safety, stability and efficacy as a biological starting material intended for a therapy or product. Stringent requirements must be inherently met, comprising negligible probability of communicable disease transmission and prolonged maintenance of a defined and differentiated phenotype. Conserved proliferation characteristics throughout industrial production passages and relatively low technical limitations are equally important (Doyle and Griffiths, 1998).

**Figure 1:**
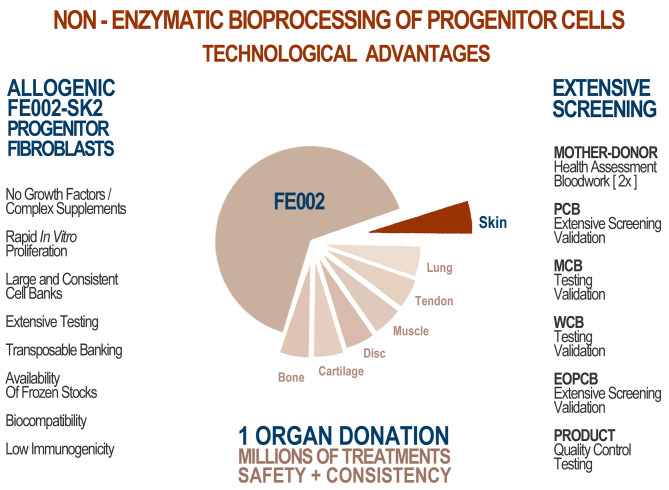
Technological advantages of appropriate whole-cell bioprocessing for skin progenitors. From one single organ donation (FE002, 2009), various samples yielding differentiated tissue-specific progenitor cells were bioprocessed for isolation using the proprietary non-enzymatic method. Intrinsic cellular identity and characteristics were therein optimally maintained throughout the transposition to adherent monolayer culture. Inherent technical and clinical advantages are attributed to the specific choice of the cell source. Optimized and consistent biobanking allowed for establishment of extensive GMP cell banks. Thorough testing throughout manufacturing along with the consistency of the cell source guarantees optimal homogeneity and safety of the biological starting material for therapeutic products. Hundreds of millions of treatments can be produced based on the available stocks.

Fetal progenitor dermal fibroblasts constitute worthy candidates in the search for the optimal cell source to be harnessed. Assuming that such cell types are properly isolated, expanded and preserved, progeny cells are reproducibly differentiated while maintaining low immunogenic properties, whereas the expansion and regenerative stimulation properties remain elevated (Quintin et al., 2007). Within consistent bioprocessing methodologies, these cells present low growth requirements (*in vitro* monolayers), are widely biocompatible with numerous natural and engineered scaffolds, are resistant to oxidative stress and are proven as effective trophic mediators of scarless wound healing (Shah et al., 1992; Cass et al., 1997; Doyle and Griffiths, 1998). Identity, purity, sterility, stability, safety and efficacy are furthermore most easily demonstrable when validating robust fetal progenitor cell banks (Figure 1) (Quintin et al., 2007).

**Figure 2:**
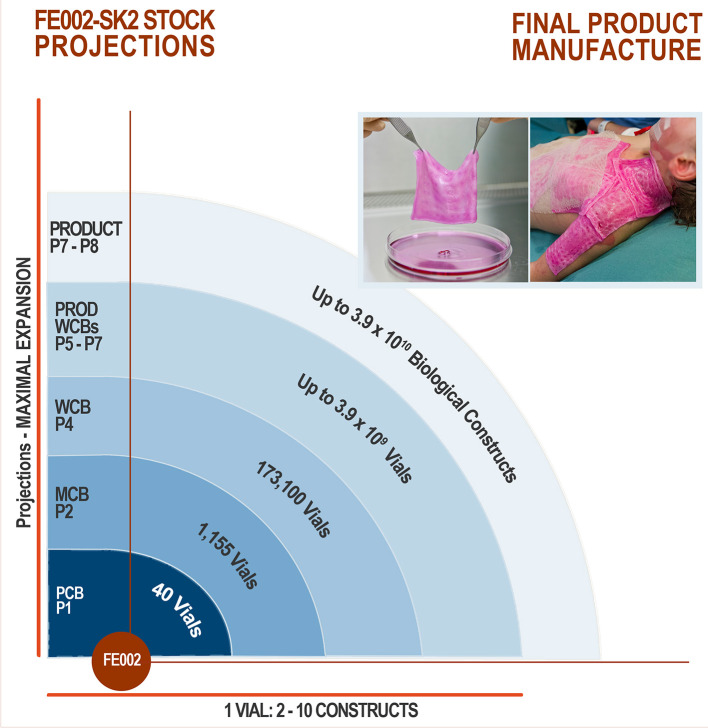
FE002-SK2 stock projections and product manufacture. Assuming maximal expansion of all available cells, the theoretical number of final products is quantified in dozens of billions. Quality and safety testing are highly consumptive in materials throughout the GMP manufacturing process, but further optimization of bioprocessing and biobanking protocols would reasonably allow for such numbers of effective treatments to be produced, using one dedicated organ donation. To manufacture end-products, cryopreserved FE002-SK2 cells at specific passages are thawed and seeded on sterile bio-compatible scaffolds, which are applied to patients following defined clinical protocols.

An optimized process for cell source selection and primary cell isolation methodology, as described herein, results in highly consistent starting biological materials for therapeutic product development, such as the non-enzymatically isolated FE002-SK2 progenitor dermal fibroblast source and progeny (Swiss transplantation laws and approved Protocols from the Lausanne University Hospital Medical Ethics Committee) (Protocol #62/07: “Development of fetal cell banks for tissue engineering,” August 2007; ECACC 12070301-FE002-SK2) (Figure 1) (Laurent-Applegate, 2012; Applegate et al., 2013). Such sources have been stringently optimized throughout tissue procurement, cellular isolation and whole-cell bioprocessing. Validated technical specifications for cell culture-expansion, biobanking and extensive testing certify consistency and safety of the progeny cell banks (Supplementary Figure 1) (Quintin et al., 2007). To this day, FE002-SK2 fibroblasts and equivalents used in “Progenitor Biological Bandages” (PBB, skin progenitor fibroblasts on an equine collagen scaffold) have been applied clinically to treat severe burn patients in the Lausanne University Hospital (CHUV) with unique results (Figure 2) (Hohlfeld et al., 2005; Ramelet et al., 2009; De Buys Roessingh et al., 2015). Most importantly, successful up-scaling and industrial transposition of the novel progenitor cell technology have allowed significant translational research to advance both in Switzerland and in Asia. The fact that, withstanding the different regulatory and technical hurdles, a single organ donation (FE002) in Switzerland in 2009 was sufficient to furnish enough material to last 10 years to date for numerous research and clinical applications around the world is of utmost interest. When fully exploiting the potential of the cell banks under consideration, projected numbers of 9 × 12 cm PBBs *per* organ donation reach >3.9 × 10^10^, when using cells at stable defined passages (Passages 7 to 8, P7-8) within their *in vitro* life-span (Figure 2) (Ramelet et al., 2009). Recent regulatory shifts have at present led to the implementation of a Priority Project (Bru_PBB) in the CHUV, devising an internal randomized clinical trial to validate the continued clinical use of PBBs. Clinical batches of FE002-SK2 progenitor fibroblasts are manufactured for the CHUV Burn Center by its own GMP-certified (SwissMedic accreditation, 2015) Cell Production Center (CPC), a reference of technical expertise.

We present herein successful up-scaling and industrial transposition of original bioprocessing and biobanking protocols for the FE002-SK2 progenitor fibroblasts. Such processes are part of the historical description of the unique conjuncture and scientific progression that led to the establishment of the Swiss Fetal Transplantation Program. Original *in vitro* and animal *in vivo* data are presented herein, demonstrating safety and activity of the FE002-SK2 progenitor fibroblasts. These results strengthen the core technical concepts relative to bioprocessing and biobanking, as experimental assays studying cell-cell interactions, cell lysates and conditioned media were devised for determination of mechanisms of action. The on-going clinical research around the world (clinical trial references: NCT02737748 and NCT03624023) focusing on the FE002-SK2 cell source will contribute synergistically to the vast local experience around such biological materials in Switzerland (De Buys Roessingh et al., 2015). The unique methodology adopted for the Swiss Fetal Transplantation Program development and the robustness of the FE002-SK2 clinical cell banks have already allowed for successful international GMP technology transfers to Europe and Asia. Through continued efforts directed at clinical translation, establishment of unified clinical protocols and product commercialization, quality and efficiency of patient care will in all probability be optimized worldwide with regard to all musculoskeletal tissues.

## Materials and Methods

### Pilot Study and Establishment of FE002-SK2 MCBs and WCBs Within GMP Standards

**Figure 3:**
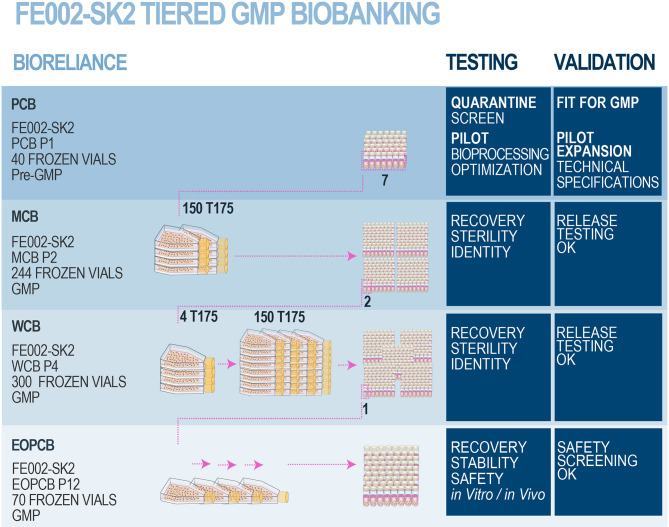
FE002-SK2 GMP biobank establishment and validation. After the FE002-SK2 PCB was declared fit for GMP manufacturing, expansion campaigns were conducted for the establishment of extensive and consistent GMP FE002-SK2 Master (P2), Working (P4), and End of Production (P12) cell banks. All the progeny cells were stored in liquid nitrogen vapor phase. Several vials of each production batch were initiated for recovery studies and quality release testing. The EOPCB materials served for extensive safety and stability assays, both *in vitro* and *in vivo*. The complete battery of tests comprised (a) isoenzyme tests, (b) sterility tests, (c) mycoplasma detection, (d) karyology, (e) transmission electron microscopy (viruses, virus-like particles, mycoplasma, yeasts, fungi, bacteria), (f) *in vitro* adventitious viruses testing (picornavirus, orthomyxovirus, pramyxovirus, herpesvirus, adenovirus, reovirus, West Nile virus), (g) *in vivo* adventitious viruses testing (suckling mice, adult mice, guinea pigs, embryonated eggs), (h) Q-PCR (HepB/C, HIV-1/2, HTLV-1/2, HHV-6/7/8, EBV, hCMV, SV40, B19 parovirus) and (i) *in vivo* tumorigenicity testing in nude mice.

After the FE002-SK2 Parental Cell Bank (PCB) was established from the FE002 organ donation in 2009 (Supplementary Figure 2), 8 PCB vials were sent frozen at −165°C to BioReliance, a GMP-certified production and storage facility (Merck Group, Glasgow, UK) for further testing (Figure 3). Initial tests were performed on quarantined vials in order to certify the admissibility of the PCB to GMP production. Stringent acceptance criteria had been defined and were also applied for the subsequent productions of tiered cell banks. The conforming assay results validated sterility of the materials, absence of bacteria, fungi, mycoplasma and viruses. Once the FE002-SK2 PCB vials were admitted for manufacturing processes, a crucial optimization phase was conducted, in order to establish the technical specifications which would be used for the creation of progeny GMP cell banks. Optimized parameters comprised culture surface size, choice of various clinical-grade substrates (growth medium, supplements) and growth conditions such as cell seeding densities and culture periods (Figure 3).

#### Establishment of a FE002-SK2 GMP Master Cell Bank

Following validated processes and cGMP (current Good Manufacturing Practices) requirements (Commission Directive 2003/94/EC), a FE002-SK2 Master Cell Bank was manufactured and validated by BioReliance (Figure 3). A total of 7 vials from the FE002-SK2 PCB (Supplementary Figure 2) stored in liquid nitrogen vapor phase were used in a recovery procedure. The cells (characterized by a viability of 97% after recovery) served for the initiation of 150 × T175 cell culture flasks (175 cm^2^, N°178883, Nunc®, USA) with a seeding density of 2.67 × 10^5^ viable cells/flask or an approximate relative seeding density of 1.5 × 10^3^ viable cells/cm^2^. The cells were cultured in DMEM (Dulbecco's Modified Eagle Medium, N°04195943M, Invitrogen™, USA) containing 10% (v/v) FBS (Fetal Bovine Serum, N°10094, Invitrogen™, USA). The culture vessels were incubated at 37°C in a humidified atmosphere with 5% (v/v) CO_2_. The culture medium was renewed every other day. The cultures were regularly photographically recorded. Cellular growth was reported as healthy (elongated fibroblast shape observed) and within specifications. One T175 flask was lost to contamination during the final medium exchange phase. After the expansion reached the predefined limit parameters (100% confluency attained in 14 days), cells at Passage 2 were harvested (D-PBS, N°14190-94, Invitrogen™, USA; Trypsin-EDTA, N°25300-062, Invitrogen™, USA), counted, resuspended in a freezing solution (DMEM:FBS:DMSO−67.5%:27.5%:5.0%, DMSO N°D2438, Sigma-Aldrich®, USA) and conditioned in individual cryovials (244 vials each containing 1.0 × 10^7^ viable cells/mL, 1.1 mL/vial, 1.8 mL capacity vials, N°368632, Nunc®, USA) to constitute the MCB. FE002-SK2 MCB vials were then frozen using a controlled-rate freezer and subsequently stored in liquid nitrogen vapor phase. Five days after the production of the MCB ended, MCB vials N°1, N°172, and N°243 were initiated to assess the recovery and cellular growth. Additional MCB vials were used in order to perform quality systems release testing. Testing of the FE002-SK2 MCB included sterility assays, mycoplasma and virus absence verification and an identity test. The MCB lot was liberated, as the samples qualified for cell viability and genetic identity, while being free of contamination by bacteria, fungi, mycoplasma, adventitious viruses, human specific viruses and bovine adventitious viruses. The total number of MCB vials produced was 244, while the number of vials released from GMP production after testing was 231.

#### Establishment of a FE002-SK2 GMP Working Cell Bank

Following validated processes and cGMP requirements, a FE002-SK2 Working Cell Bank was manufactured and validated by BioReliance (Figure 3). A total of 2 vials from the FE002-SK2 MCB stored in liquid nitrogen vapor phase were used in a recovery procedure (MCB vials N°90 and N°147). The cells (characterized by a viability of 98% after recovery) served for the initiation of 4 × T175 culture flasks (175 cm^2^, Nunc®, USA) with a seeding density of 3.52 × 10^6^ viable cells/flask or an approximate relative seeding density of 2 × 10^4^ viable cells/cm^2^. Cultures were processed in the same conditions as those of the MCB but without culture medium exchange steps. After the expansion reached the predefined limit parameters (100% confluency attained in 4 days), cells at Passage 3 were harvested as previously described, counted and used to initiate 150 × T175 culture flasks with a seeding density of 2.66 × 10^5^ viable cells/flask or an approximate relative seeding density of 1.5 × 10^3^ viable cells/cm^2^. Cultures were processed in the same conditions as those of the MCB. Cellular growth was reported as healthy (elongated fibroblast shape observed) and within specifications. No deviations occurred. After the subsequent expansion and when cultures reached the predefined limit parameters (100% confluency attained in 12 days), cells at Passage 4 were harvested, counted and conditioned in individual cryovials (300 vials containing 1.0 × 10^7^ viable cells/mL, 1.1 mL/vial) to constitute the WCB. FE002-SK2 WCB vials were then frozen as previously described and stored in liquid nitrogen vapor phase. One day after the production of the WCB ended, WCB vials N°2, N°140, and N°300 were initiated to assess cell recovery and growth. Additional FE002-SK2 WCB vials were used for the quality systems release testing. The WCB lot was liberated, as the samples qualified for cell viability and genetic identity, while being free of contamination by bacteria, fungi, mycoplasma, adventitious viruses, human specific viruses, and bovine adventitious viruses. The total number of WCB vials produced was 300, while the number of vials released from GMP production after testing was 287.

#### Establishment and Testing of an End of Production FE002-SK2 GMP Cell Bank

Following validated processes and cGMP requirements, a FE002-SK2 End of Production Cell Bank (EOPCB) was manufactured and validated by BioReliance (Figure 3). One vial from the FE002-SK2 WCB stored in liquid nitrogen vapor phase was used in a recovery procedure (WCB vial N°161). The cells (characterized by a viability of 98% after recovery) served for the initiation of 2 × T175 culture flasks (175 cm^2^, Nunc®, USA) with a seeding density of 3.53 × 10^6^ viable cells/flask. Cultures were processed in the same conditions as those of the MCB but without culture medium exchange steps. After the expansion (100% confluency attained in 4 days), cells at Passage 5 were harvested as previously described, counted and used to initiate 5 × T175 culture flasks with a seeding density of 3.45 × 10^6^ viable cells/flask. The cells were allowed to expand and serial passaging was performed in the same way using 5 × T175 flasks until confluent cultures of FE002-SK2 cells were available at Passage 11. No medium exchange steps were required and average expansion times for individual expansions were of 4 days. The harvested cells at Passage 11 were then counted and split into 30 × T175 flasks with a seeding density of 3.00 × 10^5^ viable cells/flask. Cultures were processed in the same conditions as those of the MCB and allowed to expand until 100% confluency was attained. Cellular growth was reported as healthy (elongated fibroblast shape observed) and within specifications. No deviations occurred. At the end of the final production expansion (100% confluency attained in 16 days), cells at Passage 12 were harvested, counted and conditioned in individual cryovials (70 vials containing 1.0 × 10^7^ viable cells/mL, 1.1 mL/vial) to constitute the EOPCB. FE002-SK2 EOPCB vials were then frozen as previously described and stored in liquid nitrogen vapor phase. Two days after the production of the EOPCB ended, EOPCB vials N°4, N°37, and N°67 were initiated to assess cell recovery and growth. The material from EOPCB vial N°4 was lost due to contamination and the recovery study was complemented using EOPCB vial N°5. The total number of EOPCB vials produced was 70, while the number of vials released from GMP production after testing was 66.

Tests subsequently performed on cells initiated from the FE002-SK2 EOPCB (Passage 13 after recovery) comprised (a) identification of Caucasian human origin through isoenzyme testing, (b) sterility of culture conditions, (c) mycoplasma and retroviral reverse transcriptase activity tests, (d) transmission electron microscopy (TEM) imaging with a minimum of 200 cell profiles to detect the presence of pathogens (viruses, virus-like particles, mycoplasma, fungi, yeasts, and bacteria), (e) *in vitro* testing using three control cell lines to detect viral contaminants, (f) *in vivo* testing (inapparent viruses test in suckling mice, adult mice, guinea pigs and embryonated eggs), (g) *in vivo* tumorigenicity tests in order to determine the ability of the FE002-SK2 cell type to form tumors in mice using HeLa cells as positive controls, (h) Q-PCR for B19 parvovirus screening and (i) karyotyping (Figure 3).

### Successive Technology Transfers of FE002-SK2 GMP Biobanking

**Figure 4:**
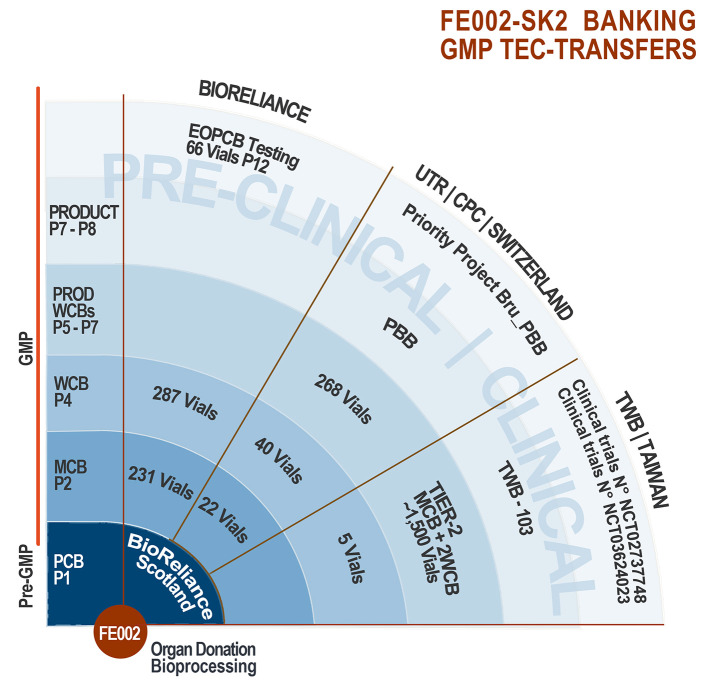
FE002-SK2 tiered biobanking and GMP tec-transfers. The robust biobanking protocols devised for the FE002-SK2 progenitor fibroblasts allowed for successful technology transfers to BioReliance (Scotland), Transwell Biotech Co. Ltd. (Taiwan), and the CHUV CPC (Epalinges/Lausanne, Switzerland). Varying banking nomenclatures exist between the different groups, the pertinent passage numbers being those used by BioReliance. Quantities of vials listed exclude those consumed for recovery studies and release testing. Pre-clinical safety testing was performed on the EOPCB by BioReliance. Clinical applications comprise the ongoing use of PBBs in the CHUV Burn Center, the pending Priority Project Bru_PBB and both clinical trials sponsored in Asia by TWB.

Following the industrial up-scaling and transposition of FE002-SK2 banking to cGMP standards performed in collaboration with BioReliance, technology transfers were operated twice successfully to date (Figure 4). In an industrial setting, part of the FE002-SK2 cell source was made available to Transwell Biotech Co. Ltd. in Taiwan (TWB), at the ITRI (Industrial Technology and Research Institute) GMP facility in order to establish Tier-2 Working Cell Banks (“Tier-2 WCB”). Furthermore, cells from the Tier-2 WCBs were reinitiated, culture-expanded and cryopreserved as a component of the final TWB therapeutic product (FE002-SK2 progenitor fibroblasts renamed “TWB-102 cells” in a hydrogel scaffold) for preclinical research and clinical trials. Three co-Authors (BS, CS, LAA) oversaw the transmission of scientific and technical know-how in Taiwan. In a hospital/clinical setting, part of the FE002-SK2 cell source was donated to the CHUV Burn Center, represented by the state-of-the-art CPC for manufacturing purposes, for the continuation of burn patient care using Progenitor Biological Bandages. Manufacturing protocols were transposed to the CPC and subsequently internally optimized. Although FE002-SK2 banking nomenclatures vary between the different GMP production sites around the world, the same original stock of biological material (FE002-SK2 PCB) served as a source for the incremental development of all clinical-grade cell banks. Pre-GMP FE002 tissue donation bioprocessing and FE002-SK2 PCB establishment in the CHUV accredited laboratory under the Transplantation Program enabled the transition to full GMP banking, which was thereafter repeatedly and successfully transposed (Figure 4). This in turn allowed for the continuation of applied research and development related to the FE002-SK2 cell source of interest, both *in vitro* and *in vivo*, for which selected original data are presented hereafter.

### *In vitro* Effects and *in vivo* Safety of FE002-SK2 Progenitor Fibroblasts

#### Transwell® Migration Assay of Primary Keratinocytes in Presence of FE002-SK2 Progenitor Fibroblasts

Sterile Transwell® inserts (6.5 mm diameter polycarbonate membranes with 8 μm pores, N°3422, Corning®, USA) and 24-well cell culture plates (N°353047, Corning®, USA) served as the experimental scaffold. In each assay repetition, triplicate wells were cultured in parallel for each study group. On Day 0 and for the test group, culture wells were seeded with 9 × 10^4^ FE002-SK2 cells at P6 suspended in 0.5 mL of DMEM (N°11995, Gibco®, USA) supplemented with 10% FBS (N°10091, Gibco®, USA). Cell counting was performed using an ADAM-MC™ cell counter (NanoEnTek, South Korea). For the control group, the same culture medium without cells was added appropriately to additional wells. Cultures were incubated overnight at 37°C and under 5% CO_2_. On Day 1, all media were removed and the culture wells were gently washed twice with DPBS (N°14190, Gibco®, USA), followed by the addition of 0.5 mL of K-SFM (Keratinocyte Serum-Free Medium, N°17005-042, ThermoFisher, USA) to each well. A sterile Transwell® was inserted into each well used in the assay. In the upper part of each Transwell®, 6 × 10^4^ primary human foreskin keratinocytes (NHEK-Neo, N°00192906, Lonza, Switzerland, P3 or P4 at use) were then seeded, suspended in 0.3 mL of K-SFM. The culture vessels were transferred to a humidified incubator set at 37°C and 5% CO_2_ for 15–18-h. On Day 2, after removal of the media from the Transwells®, the inserts were immersed in a 10% paraformaldehyde solution (N°16005, Sigma-Aldrich®, Germany) for 30 min. Thereafter, the Transwells® were immersed in a 1% crystal violet solution (N°V5265, Sigma-Aldrich®, USA) for 30 min for cell-staining. The inserts were then rinsed with sterile water and the keratinocytes which remained on the upper sides of the membranes were removed gently using cotton swabs. The keratinocytes which had migrated toward the lower sides of the membranes were further stained with DAPI (4′,6-diamidino-2-phenylindole, N°D8417, Sigma-Aldrich®, USA). Finally, after washing, the DAPI-stained cells on the membranes were visualized by fluorescence microscopy using excitation wavelengths 340–390 nm. Under 100X magnification, 5 photos were taken for each membrane including the upper, lower, left, right, and center fields. The photos were analyzed using ImageJ (NIH, USA). Cell counts (DAPI spots) on all 5 photos were accumulated. Mean total cell counts from the test groups were compared to those of the control groups.

#### Gap-Filling Assay Using Primary Keratinocytes in Presence of Media Conditioned by FE002-SK2 Progenitor Fibroblasts

The Culture-Insert 2 well in μ-Dish 35 mm (N°81176, ibidi®, Germany) served as an experimental scaffold. The device comprised a 35 mm dish with an insert disposed in its middle. The insert included 2 wells separated by a 0.5 mm wide divider. These wells were coated with collagen (rat tail collagen, N°354236, Corning®, USA, diluted with 0.02 N HCl to 50 μg/mL for coating) before use for the assay. To prepare FE002-SK2 cell conditioned media, the cells (P8) were grown in 6-well culture plates (N°140675, Nunc®, USA) in DMEM supplemented with 10% FBS (N°10091, Gibco®, USA) in a humidified incubator set at 37°C and 5% CO_2_ until 70–100% confluency was attainted. After media removal and two washes with DPBS, 2.5 mL of K-SFM were added to each well. The plates were re-incubated as mentioned hereabove. Conditioned media were collected 24-h later and processed using a 0.22 μm syringe filter (N°LGP033RB, Millipore, Germany) before direct use in assays or storage at −80°C until use. The sham medium was prepared following an identical process, except that no cells were present in culture. For the experimental assay, 2.25–4.20 × 10^4^ primary human foreskin keratinocytes (N°881122-01-K, ATRI, Taiwan, P4 or P5 at use) suspended in fresh K-SFM were seeded in both wells of the ibidi® insert (1.0–1.9 × 10^5^ keratinocytes/cm^2^). An additional 1 mL of fresh K-SFM was added to the dish area outside the insert and the device was incubated at 37°C with 5% CO_2_. After 17-h of culture, 70 μL of test medium (conditioned medium or sham medium) were dispensed inside the wells to replace the K-SFM. The device was incubated at 37°C for another 6-h to allow the keratinocytes to respond to the test media. To initiate cell migration, the medium outside the insert was also replaced by test media and the insert was removed from the dish. At that moment, a 0.5 × 7.0 mm gap devoid of cells was created between areas of confluent keratinocytes. The assay plates were re-incubated, the keratinocytes being free to migrate. Representative imaging was performed at time points of 0, 3, 6, 9, 12, 15, and 18-h after initiation of migration. The resulting composed images were analyzed using ImageJ to integrate the area of cell migration.

#### Proliferation of Primary Keratinocytes in the Presence of FE002-SK2 Cell Extracts

Thawed suspensions of FE002-SK2 cells (P8, 7.5 × 10^6^ cells/mL in a DMEM-based solution) were sequentially centrifuged at 20,600 × g for 10, 5, and 3 min, with the pellets being resuspended in the original medium after the first and second centrifugations. After the third centrifugation, supernatants were collected and defined as cell extracts. No cells were observed after cell extracts were put in culture (DMEM, 10% FBS). On Day 0, 1.92 × 10^4^ keratinocytes (NHEK-Neo) suspended in 2 mL of DK-SFM (Defined Keratinocyte Serum-Free Medium, N°10785-012, Gibco®, USA) were seeded in each of the collagen-coated wells of 6-well culture plates. FE002-SK2 cell extracts were then added to the keratinocyte cultures in the amount of 0, 19.2, or 38.4 μL/well at the defined timepoints. Cultures were incubated at 37°C and under 5% CO_2_. Cell extracts in appropriate doses were added once (on Day 0) or twice (on Day 0 and Day 2) over the course of the assay. The culture medium was changed on Day 2 (before adding the extracts) and on Day 5. On Day 7, the cells were detached (TrypLE™, N°12563, Gibco®, USA) and counted.

#### Proliferation of Primary Keratinocytes in the Presence of Mitomycin C-Treated FE002-SK2 Progenitor Fibroblasts

Mitomycin C (N°M4287, Sigma-Aldrich®, USA) was dissolved in DMSO (N°D2438, Sigma-Aldrich®, USA) to constitute a 1 mg/mL stock solution. FE002-SK2 cells at P10 were cultured in DMEM supplemented with 10% FBS in the same conditions as previously described until 90% confluency was attained. Mitomycin C stock solution was added to cultures by dilution in the growth medium, targeting a final concentration of 5 μg/mL. Cultures were re-incubated for 1-h. The supplemented medium was then removed and mitomycin C-treated FE002-SK2 cells were washed thrice with DPBS, trypsinized (Trypsin-EDTA, N°25300, Gibco®, USA), collected by centrifugation (7 min, 230 × g) and cryopreserved as previously described. After thawing, these cells attached to the culture vessels but did not proliferate. Both uncoated and collagen-coated 6-well culture plates (N°140675, Nunc®, USA) were used as scaffolds to assess the proliferation of primary keratinocytes (NHEK-Neo) in the presence of mitomycin C-treated progenitor fibroblasts (MMC-FE002-SK2). For the experimental assays, 1.92 × 10^4^ keratinocytes suspended in DK-SFM were seeded in each well of the 6-well culture plates (2,000 cells/cm^2^) and cultured as previously described for several hours until fully attached. Then, MMC-FE002-SK2 cells were thawed, washed, suspended in DK-SFM and seeded in the same wells at 2.90 × 10^4^ cells/well (3,000 cells/cm^2^) and the cultures were maintained for another 6 days. The media were renewed twice during this period. Wells containing only keratinocytes or only MMC-FE002-SK2 cells served as controls. Cell proliferation was monitored and photographically recorded. Proliferating cells in the co-cultures were verified as keratinocytes based on immunostaining using anti-cytokeratin-14 (CK-14) antibodies. Briefly, cultures in the 6-well plates were washed with DPBS and fixed with 4% paraformaldehyde. After cold DPBS wash and permeabilization of cells with 0.1% Triton™ X-100 (N°X100, Sigma-Aldrich®, USA), the wells were blocked with 1% bovine serum albumin (BSA, N°A7030, Sigma-Aldrich®, USA) at room temperature for 30 min. Then, 200X-diluted anti-CK-14 mouse antibodies (N°ab7800, Abcam, UK) were added to the wells and plates were incubated for 1-h at room temperature. The wells were then washed 3 times with DPBS. 1,000X-diluted Alexa Fluor 488 goat anti-mouse (H+L) antibodies (N°A-28175, ThermoFisher, USA) were added and plates were incubated at room temperature for 1-h. After 3 successive DPBS washes, wells were observed and recorded using appropriate fluorescence microscopy (excitation wavelengths filter 471–495 nm).

#### Proliferation of Primary Keratinocytes in Co-culture With FE002-SK2 Progenitor Fibroblasts and Biomarker Analysis of Conditioned Media

Cryopreserved keratinocyte suspensions (NHEK-Neo, P3) were thawed, diluted with DK-SFM, centrifuged and cells were resuspended in fresh DK-SFM. For the experimental assays, 1.92 × 10^4^ keratinocytes suspended in DK-SFM were seeded in each well of collagen-coated 6-well culture plates (N°140675, Nunc®, USA, 2,000 cells/cm^2^) and cultured for several hours as previously described until fully attached. Cryopreserved FE002-SK2 cells (P8, in non-DMSO cryopreservation solution) were thawed and diluted with DK-SFM before seeding at 4.80 × 10^4^ cells/well (5,000 cells/cm^2^) or 5.76 × 10^4^ cells/well (6,000 cells/cm^2^) on the same 6-well plates and cultures were maintained. Additional wells were prepared appropriately for controls. All wells were then supplemented with additional DK-SFM to the total volume of 2 mL. On Day 3 and Day 5, the media in all wells were replaced with 2 mL of fresh DK-SFM. On Day 6, the media (which had been conditioned for 24-h) were collected before the cells were washed, detached and counted. Conditioned media were collected from the cultures of FE002-SK2 cells (P9) and keratinocytes (P4) for biomarker analysis. These samples, along with the media collected from co-cultures on Day 6, were centrifuged at 805 × g for 5 min at 4°C. The supernatants were collected and kept at −80°C before being transported to RayBiotech Life (USA) for analysis by “Human 200 Biomarker Testing”. There were 200 protein factors analyzed, including growth factors, cytokines, cell adhesion factors, ECM proteins and enzymatic modulators. Each factor was quantified using a reference curve.

#### GLP Porcine Study Using FE002-SK2 Progenitor Fibroblasts for Split-Thickness Wounds

The purpose of *in vivo* testing was to primarily evaluate safety of application of FE002-SK2 progenitor fibroblasts in treating split-thickness wounds (excision wound) on a porcine model. The primary goals were to evaluate whether a defined cell-based product caused adverse effects on wounds or peri-wound tissues, hindered wound healing, or caused adverse effects on general health of animals. The study was carried out, after proper ethical considerations were validated at the Agricultural Technology Research Institute in Taiwan, on 5 male domestic pigs (Landrace cross). Four test articles were investigated (sham control [A], hydrogel scaffold alone [B] and hydrogel scaffolds with low [C] and high doses [D] of progenitor fibroblasts). Four wounds were created on the dorsum of each pig. Using a dermatome (N°8821-01, Zimmer®, USA), square wounds (5.1 ± 1.0 cm sides, 0.45 ± 0.15 mm deep) were created on areas which were 4 cm distant from the spine. Two wounds were created on each dorsum side and were separated from each other by 5 cm. Every pig received four kinds of test articles (A-D), with one wound receiving one kind of test article (A, B, C, or D). There were 5 wounds for each group of test articles (*n* = 5 in each group). Each wound was treated with the respective test article on Days 0 (wound creation date), 3, 6, and 9. The wounds were then covered with 3M^TM^ Tegaderm^TM^ (3M, USA, waterproof and gas-permeable film gauze, 6 × 7 cm). Measurements of wound area and photographic recording were carried out on Days 0, 3, 6, 9, 13, and 14. Blood samples were drawn on Days 0, 3, and 14 and processed for hematology and biochemistry examinations. On Day 14, the skin samples on the original wound and peri-wound areas were excised (7.6 ± 1.0 cm sides, 0.60 ± 0.15 mm deep) and soaked in normal saline to rinse off blood. A half of the biopsy was soak-fixed in 10% (v/v) neutral formalin solution, while the other half was stored at −80°C. Tissue sections (6 μm) were stained using hematoxylin and eosin (HE) and histologically examined. Animals were sacrificed immediately after skin biopsies were harvested.

## Results

### Pilot Study and Establishment of FE002-SK2 MCBs and WCBs Within GMP Standards

Pre-GMP FE002 tissue donation bioprocessing and FE002-SK2 PCB establishment had been performed in the CHUV accredited laboratory under the Swiss Fetal Transplantation Program. FE002-SK2 progenitor fibroblasts had been extensively characterized in the same manner as the previous cell sources used in anterior successive Transplantation Programs (De Buys Roessingh et al., 2006; Quintin et al., 2007; Applegate et al., 2009; Laurent-Applegate, 2012). Extensive optimization steps, additional screening and close collaboration allowed for transposition of the biological materials and banking procedures to cGMP standards in BioReliance (Figures 3, 4). Large and consistent GMP MCB (244 vials, cells at Passage 2), WCB (300 vials, cells at Passage 4) and EOPCB (70 vials, cells at Passage 12) were manufactured and qualified, based on a unified source and serving for further international research and clinical developments (Figure 4). Most importantly, in view of clinical applications, the FE002-SK2 progeny cell banks were extensively tested, and results fell within predefined validated specifications for all assays. Release testing was performed for FE002-SK2 Master and Working Cell Banks, while extensive *in vitro* and *in vivo* safety testing assays were performed on the EOPCB (Figure 3).

Screening tests that were accomplished on the FE002-SK2 EOPCB (cells at Passage 13 after recovery) confirmed the identification of Caucasian human origin through isoenzyme testing. Sterility of culture conditions was also confirmed. Mycoplasma and retroviral reverse transcriptase activity tests were negative. Transmission electron microscopy (TEM) imaging with a minimum of 200 cell profiles to detect the presence of pathogens (viruses, virus-like particles, mycoplasma, fungi, yeasts and bacteria) was accomplished and all test-results were documented as negative (data not shown). No contaminants were detected using all other assays including (a) *in vitro* testing using three control cell lines to detect viral contaminants, (b) *in vivo* testing (inapparent viruses test in suckling mice, adult mice, guinea pigs, and embryonated eggs) (100% survival was obtained for all the tests) and (c) Q-PCR for B19 parvovirus screening. *In vivo* tumorigenicity testing was performed in order to determine the ability of the FE002-SK2 cell type to form tumors in mice using HeLa cells as positive controls. Results indicated that no tumorigenicity was evident. Karyotyping was also accomplished to assess cell stability throughout passages and 50 metaphases were examined. No polyploidy was observed and no chromosome aberrations were noted. For each cell passage from Passage 5 to Passage 12, the viability from the recovery studies ranged from 98 to 100% with mean doubling times ranging from 37.5 to 71.6-h (P6-37.5 h; P7-46.9 h; P8-52.2 h; P9-47.5 h; P10-51.1 h; P11-71.6 h; P12-65.1 h) (data from FE002-SK2 EOPCB establishment). Furthermore, no contamination was found during all the processing except for one vial in one recovery study and one culture flask in a medium exchange procedure.

### FE002-SK2 GMP Tec-Transfers and Product Development

Based on the results obtained during the successive GMP campaigns in BioReliance, the FE002-SK2 banking technology was transposed to TWB in Taiwan and to the CHUV Burn Center, represented for manufacturing purposes by the CPC (Figure 4). Continued development and parallel optimization steps were carried out between BioReliance and the UTR (Regenerative Therapy Unit, Applegate group) in the CHUV, to devise the most rational and efficient use of the biological materials for therapeutic application. Following sub-licensing of the technology to TWB in Taiwan, extensive product development (TWB-103 product) led to two registered clinical trials for donor site complications (“TWB-103 for Adult Patients with Split-Thickness Skin Graft Donor Site Wounds,” NCT02737748, Japan and Taiwan) and for diabetic foot ulcers (“TWB-103 for Treating Lower Limb Ulcers on Patients With DM,” NCT03624023, Taiwan). Following the long submission process, regulatory approval was therefore successfully obtained in both Taiwan (Taiwan Food and Drug Administration-TFDA) and Japan (Pharmaceuticals and Medical Devices Agency-PMDA) with associated documentation of Investigational Medicinal Product Dossiers and Investigator Brochures along with Clinical Trial documentation for monitoring. Part of the FE002-SK2 cell source was donated to the CHUV Burn Center, to continue clinical applications of Progenitor Biological Bandages (on-going in-house clinical practice and experience of 30 years) within a Priority Project (Bru_PBB).

### *In vitro* Mechanism of Action Study and *in vivo* Preclinical Safety Evaluation of FE002-SK2 Progenitor Fibroblasts

#### FE002-SK2 Progenitor Fibroblasts Facilitate Keratinocyte Migration

**Figure 5:**
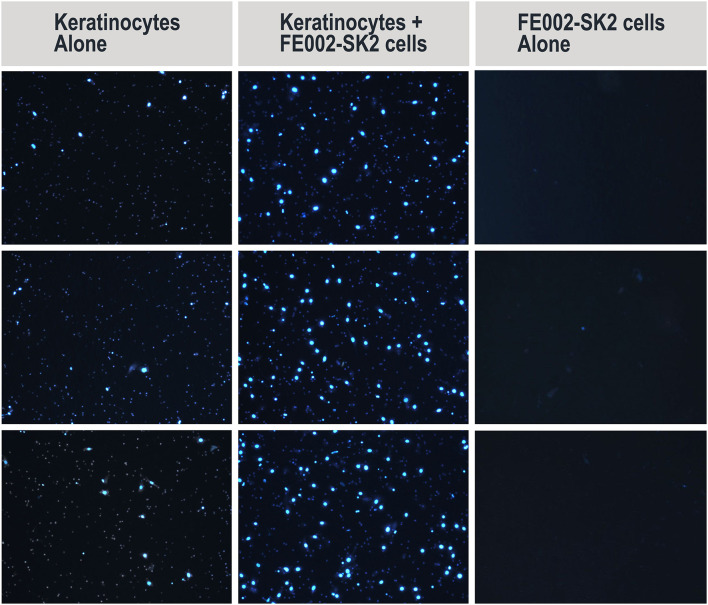
Representative imaging of migrated primary human keratinocytes (DAPI staining) in Transwell® migration assay (left and middle columns). Representative imaging includes middle fields of 3 wells/group. Contamination of Transwells® by FE002-SK2 progenitor fibroblasts was negligible (right column). Mean cell counts are listed in Table 1.

Keratinocyte migration is critical for wound healing (Spiekstra et al., 2007). To test whether FE002-SK2 cells could promote keratinocyte migration, primary human keratinocytes from mixed donors were seeded on the upper side of Transwell® membranes in 24-well culture plates. It was found that the presence of FE002-SK2 fibroblasts in the culture wells increased the number of migrated keratinocytes (Figure 5, representative imaging, DAPI staining and Table 1). The relative increase factors for migrated populations of keratinocytes under the influence of FE002-SK2 progenitor fibroblasts ranged from 1.8 to 9.3 among 15 consecutive assays (mean value of 4.4 ± SD 2.4). It was found that the passage number and source of keratinocytes affected the baseline migration rate, which resulted in considerable inter-assay variation. However, the intra-assay coefficient of variation between triplicate wells was usually within 20%. The results of a series of three assays are presented in Table 1. Contamination of Transwells® by FE002-SK2 fibroblasts was negligible.

**Table 1:** Average counts of migrated primary keratinocytes in Transwell® migration assay, in the absence and presence of FE002-SK2 progenitor fibroblasts in the culture wells.

	**A: Keratinocytes alone**	**B: Keratinocytes under influence of FE002-SK2 progenitor fibroblasts**	**Mean relative increase factor**
Exp 1	88 ± 25 (28.0%)	238 ± 30 (12.8%)	2.71
Exp 2	152 ± 18 (11.7%)	409 ± 16 (3.9%)	2.70
Exp 3	113 ± 19 (16.8%)	299 ± 33 (10.9%)	2.64

*Keratinocytes migrated downward through Transwell® membranes. Mean counts are presented for triplicates and three experimental repetitions (n = 3), with associated standard deviations and coefficients of variation [%]. Mean counts represent the sums of migrated keratinocytes in 5 integrated fields of each well. Cells were counted based on DAPI staining. Mean relative increase factors of migrated population counts are provided in the last column*.

#### FE002-SK2 Cell-Conditioned Medium Facilitates Keratinocyte Migration

**Figure 6:**
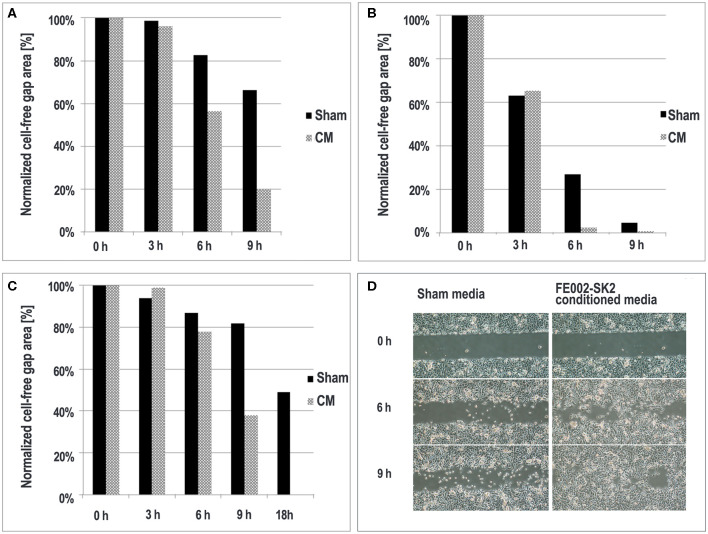
Epithelial migration in the presence of FE002-SK2 conditioned media and sham media. Quantitative results of three experimental repetitions are compared **(A–C)**. At the 9- and 18-h timepoints in the second and third replicates, respectively **(B,C)**, the gaps treated with FE002-SK2 conditioned media were completely filled. Representative imaging of epithelial migration from the first experiment is shown **(D)**. Negligible filling was detectable at the 3-h timepoint. Imaging data was acquired under 40X optical magnification.

Wound-healing is typically modeled by closing of epithelial gaps developed *in vitro* (Pastar et al., 2014). The ability of FE002-SK2 conditioned medium to facilitate epithelial cell migration was tested using primary keratinocytes in gap-filling assays. The results showed that the rate of gap-filling was higher in the group using FE002-SK2 conditioned media than in the group using sham media (Figure 6). Adding conditioned medium promoted faster closing of the epithelial gaps, when considering appropriate timepoints and under specific experimental conditions. In three considered experimental repetitions, a significant difference in migration area covered by the keratinocytes was observed after 6–9-h of incubation. In this assay, before the initiation of migration (i.e., the time when the divider between two patches of confluent keratinocytes is removed) the keratinocytes must be preincubated with the test media to be converted to the fast-migrating type. Without the pre-incubation, the gap would be filled in both sham and test media before differential migration speeds can be detected (data not shown). Representative imaging of gap-filling at timepoints between 0- and 9-h are shown in Figure 6D.

#### FE002-SK2 Cell Extracts Promote Keratinocyte Proliferation

**Figure 7:**
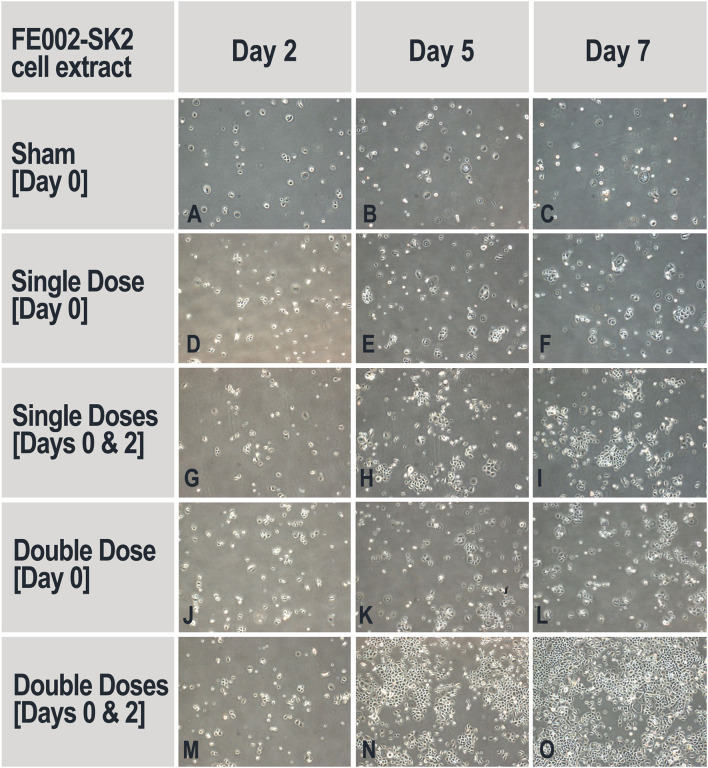
Representative imaging of keratinocyte proliferation in the absence and presence of FE002-SK2 cell extracts. Adjunction of sham medium or cell extracts on Day 0 did not stimulate keratinocyte proliferation and did not ensure maintenance of cell populations **(A–F,J–L)**. Adjunction of cell extracts on Day 0 and Day 2 resulted in augmented keratinocyte proliferation **(H,I,N,O)**. Imaging data was acquired under 40X optical magnification. Mean harvesting total cell counts are listed in Table 2.

Keratinocyte proliferation is another critical factor for wound healing (Usui et al., 2008). The potential of FE002-SK2 cell extracts to promote keratinocyte proliferation was assessed, simulating the liberation of active biochemical factors after cell lysis on the patient or in therapeutic constructs. It was found that without the addition of progenitor cell extract, the primary keratinocyte population was reduced after the culture period, indicating that the medium did not support cell maintenance and growth (Table 2). It was required to add the cell extracts twice, on both Day 0 and Day 2 to stimulate keratinocyte proliferation (Figure 7). Adding 38.4 μL of extract twice clearly and extensively promoted keratinocyte proliferation (Figures 7N,O). Multiple adjunctions of cell extract consistently resulted in relatively more potent stimulation of keratinocyte proliferation. In this assay and in the proliferation assays in the following sections, the media used were DK-SFM. This medium had lost its ability to support keratinocyte growth after a short period of appropriate storage, even prior to the indicated expiration date.

**Table 2:** Primary keratinocyte proliferation represented by mean total population counts in the absence and presence of FE002-SK2 cell extracts.

**FE002-SK2 cell extract dosing regimen**	**Viable keratinocyte mean counts [10**^****4****^ **cells/well] on Day 7 (Dosed/Sham)**		
	**Exp 1**	**Exp 2**	**Exp 3**
Sham Day 0	0.094 (1.0)	0.48 (1.0)	2.22 (1.0)
Single Dose Day 0	0.69 (7.3)	1.24 (2.6)	3.92 (1.8)
Single Doses Day 0 and Day 2	2.41 (25.6)	3.25 (6.8)	9.45 (4.3)
Double Dose Day 0	1.55 (16.5)	1.33 (2.8)	4.53 (2.0)
Double Doses Day 0 and Day 2	19.60 (208.5)	7.68 (16)	12.28 (5.5)

“*Sham” conditions corresponded to adding 0 μL of extract, “Single Dose” conditions corresponded to adding 19.2 μL of extract and “Double Dose” conditions corresponded to adding 38.4 μL of extract. The initial viable keratinocyte mean count (Day 0) was of 1.92 × 10^4^ cells/well. Mean counts are presented for duplicates and three experimental repetitions (n = 3). Ratios corresponding to total viable cell counts from the dosed wells over those of the sham wells on Day 7 are presented in parentheses (Dosed/Sham)*.

#### Mitomycin C-Treated FE002-SK2 Progenitor Fibroblasts Promote Keratinocyte Proliferation

**Figure 8:**
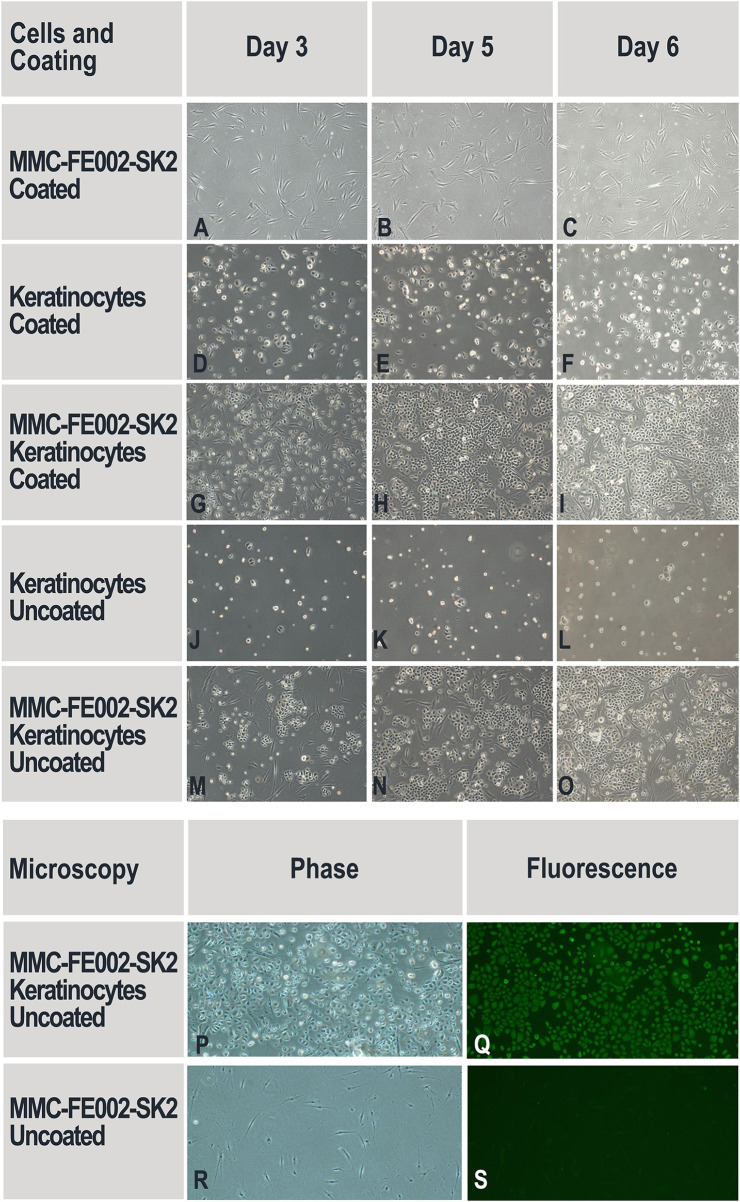
Proliferation of primary keratinocytes in the presence of mitomycin C-treated FE002-SK2 progenitor fibroblasts (MMC-FE002-SK2 cells). DK-SFM is deficient in extracellular matrix (ECM) proteins. Considering that FE002-SK2 fibroblasts can secrete ECM proteins for cell attachment, both collagen-coated **(A–I)** and un-coated surfaces **(J–S)** were comparatively tested. No cell proliferation was observed in the single-population cultures of keratinocytes **(D–F,J–L)** or MMC-FE002-SK2 cells **(A–C)**. Keratinocyte proliferation was observable in co-cultures **(G–I,M–P)**. Selected wells were also stained with anti-CK-14 antibodies **(P–S)**. MMC-FE002-SK2 progenitor fibroblasts appeared negative for immunostaining **(S)**, while proliferating cells in co-culture wells appeared positive **(Q)**. Staining data confirmed that proliferating cells were keratinocytes. Imaging data was acquired under 40X optical magnification and fluorescence microscopy.

Although FE002-SK2 cell extracts were capable of promoting keratinocyte proliferation, multiple adjunctions were required for continuous and effective stimulation. The potential for proliferation stimulation of integral and growth-arrested FE002-SK2 cells (MMC-FE002-SK2 cells) constantly interacting with primary human keratinocytes was assessed. The results showed that in the absence of MMC-FE002-SK2 cells, keratinocytes did not proliferate in either collagen-coated or un-coated wells (Figures 8D–F,J–L). MMC-FE002-SK2 cells did not proliferate but were able to promote keratinocyte proliferation in co-cultures already since Day 3. The proliferation promotion was evident in both the coated and un-coated wells (Figures 8G–I,M–O). The cells that proliferated in the co-culture wells verified to be keratinocytes based on immunostaining (Figures 8Q,S) and the MMC-FE002-SK2 cells were verified to have not proliferated.

#### Proliferation of Keratinocytes in Co-culture With FE002-SK2 Cells and Biomarker Analysis of Conditioned Media

**Figure 9:**
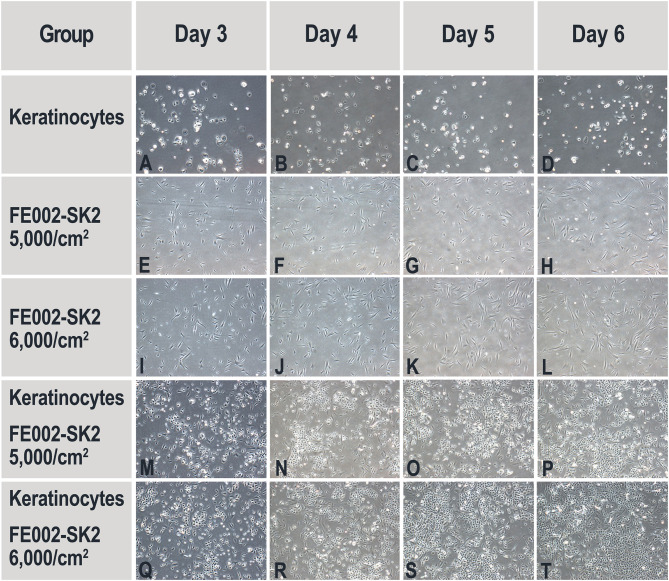
Representative imaging of primary keratinocyte and FE002-SK2 progenitor fibroblast proliferation in single- and co-cultures. Initial viable seeding densities are indicated for each FE002-SK2 group. Initial viable seeding density of keratinocyte was of 2,000 cells/cm^2^. No proliferation was observed in the single-population cultures of keratinocytes **(A–D)** or FE002-SK2 fibroblasts **(E–L)**. High proliferation was observable in the co-cultures **(M–T)**. Imaging data was acquired under 40X optical magnification. Mean harvesting cumulated cell counts are listed in Table 3.

The results presented hereabove indicated that keratinocyte migration and proliferation were activated by the FE002-SK2 cells or derivatives thereof. In order to study the factors involved in such activations, co-culture experiments were carried out. Table 3 shows that neither pure FE002-SK2 nor keratinocyte populations proliferated over the 6-day culture period. However, in the presence of FE002-SK2 cells, keratinocytes grew to large colonies (Figures 9M–T, Table 3). Conditioned media biomarker analysis results are presented in Table 4. For the DK-SFM control samples, the only factors detected at a significant level (defined as 3-fold of the lower Limit Of Detection, LOD) were insulin and low levels of LIF (Table 4). EGF was not detected. For the keratinocyte conditioned media, 14 factors were detected (CXCL16, E-Selectin, Follistatin, Galectin-7, IGFBP2, Insulin, LIF, Lipocalin-2, MIF, PAI-1, PDGF-AA, TIMP1, TIMP2, and Trappin-2). For the FE002-SK2 conditioned media, 12 factors were detected (Angiogenin, CD14, CEACAM1, GDF15, HGF, IGFBP6, Insulin, LIF, Lipocalin-2, PAI-1, TIMP1, and TIMP2). For the co-culture media, several additional factors were detected. Table 4 lists the factors which were significantly present in at least one of the cell conditioned media. Many of the factors were present at relatively high levels in the co-culture media but at low levels in the media from single population cultures. Galectin-7 was present at high levels in the keratinocyte conditioned media but the concentration was relatively reduced about 5-fold in the co-culture media (Table 4).

**Table 3:** Primary keratinocyte and FE002-SK2 progenitor fibroblast proliferation in co-cultures.

**Cells seeded on Day 0**		**Exp 1**	**Exp 2**	**Exp 3**
**Keratinocytes [10^**4**^ cells/well]**	**FE002-SK2 fibroblasts [10^**4**^ cells/well]**	**Mean viable cumulated cell counts on Day 6 [10^4^ cells/well]** **(Day 6/Day 0)**	**Mean viable cumulated cell counts on Day 7 [10^4^ cells/well]** **(Day 7/Day 0)**	**Mean viable cumulated cell counts on Day 7** **[10^4^ cells/well]** **(Day 7/Day 0)**
1.92	0	0.88 (0.46)	0.23 (0.12)	0.27 (0.14)
0	4.80	1.65 (0.34)	2.54 (0.53)	1.99 (0.41)
0	5.76	2.15 (0.37)	4.34 (0.75)	3.36 (0.58)
1.92	4.80	41.60 (6.19)	8.35 (1.24)	31.60 (4.70)
1.92	5.76	47.78 (6.22)	10.78 (1.40)	33.93 (4.42)

*Mean cumulated viable counts are presented for duplicates and three experimental repetitions (n = 3). Ratios corresponding to cumulated viable cell counts from the dosed wells on Day 6 or Day 7 over initial cell counts in those same wells on Day 0 are presented in parentheses (Day 6–7/Day 0)*.

**Table 4:** Biomarkers detected in the control media, in single population cultures and in keratinocyte-FE002-SK2 co-culture conditioned media.

	**LOD**	**DK-SFM medium after incubation**	**DK-SFM from keratinocyte cultures**	**DK-SFM from FE002-SK2 cultures**	**DK-SFM from co-cultures**
Angiogenin	3	0	1	9	**42**
AR	25	0	51	0	**645**
Axl	13	11	21	27	**76**
CD14	11	8	0	210	12^*^
CEACAM1	13	26	23	73	5^*^
CXCL1	5	0	1	1	**105**
CXCL5	12	2	8	4	**3,499**
CXCL8	7	11	12	7	**448**
CXCL16	12	0	41	0	**502**
DKK-1	73	0	0	0	**280**
DR6	4	0	6	0	**72**
EGFR	8	0	6	1	**40**
E-Selectin	80	0	306	0	**608**
Fas	3	0	0	0	**14**
Follistatin	21	3	103	35	**1,453**
Galactin-7	103	0	2,672	0	588^*^
GCP-2	11	0	0	0	**75**
GDF15	2	0	1	6	**72**
HGF	12	8	5	41	**119**
IGFBP2	23	0	192	0	**1,660**
IGFBP6	241	0	0	3,452	**5,945**
IL-6	17	23	7	25	**2,383**
IL-6R	113	0	0	7	**468**
Insulin	55	11,947	14,882	9,988	9,964
LIF	18	58	56	118	146
Lipocalin-2	1	0	132	3	**429**
MCP-1	25	0	0	0	**382**
MCSF	2	0	0	0	**11**
MIF	15	0	271	27	155^*^
MIP-3a	1	0	0	0	**17**
MIP-1b	3	0	1	1	**23**
PAI-1	115	0	1,657	13,922	**19,550**
PDGF-AA	3	6	14	0	**40**
PIGF	3	3	1	3	**12**
RANTES	11	2	12	2	**225**
TGFα	6	0	1	0	**35**
TIMP1	23	15	247	6,587	**5,891**
TIMP2	26	9	135	5,098	**4,443**
TNFα	25	21	58	43	76
TNF-R1	24	14	42	27	**274**
Trappin-2	21	0	708	0	**961**
VEGF	8	0	3	0	**105**

*Concentration values as well as LODs (lower limits of detection) are presented in [pg/mL]. Values having significantly increased from the single population cultures to the co-cultures are presented in bold in the co-culture column. Values having significantly decreased from the single population cultures to the co-cultures are presented with an asterisk in the co-culture column. Significant increases were defined as levels beyond an additive resulting effect in the co-cultures, normalized to the final mean cumulated cell counts*.

#### Porcine *in vivo* Study on Standardized Wounds

**Figure 10:**
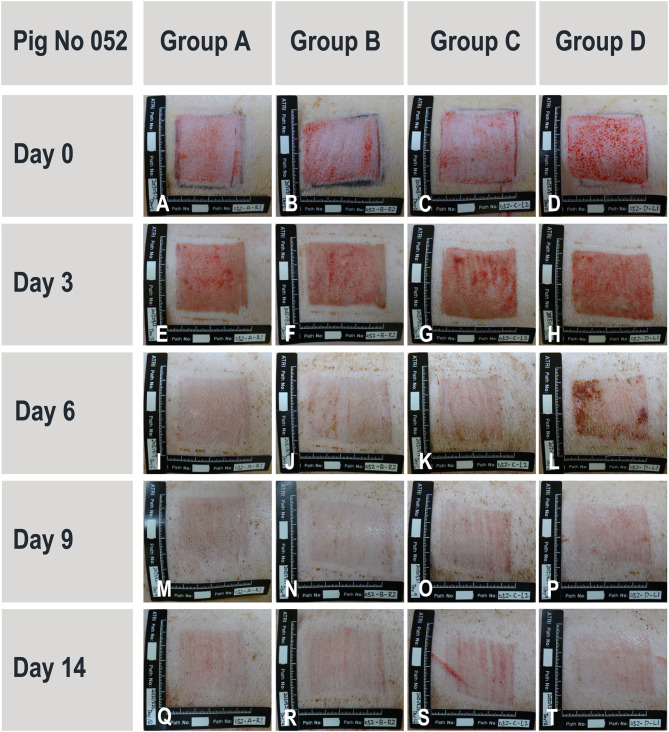
Representative and evolutive imaging of porcine wound models. Data were acquired from pig N°052. All 4 experimental groups are presented at different timepoints ranging from wound creation (Day 0) up until sacrifice (Day 14). Treatment groups [A–D] consisted in the sham control, hydrogel scaffold alone, and hydrogel scaffolds with low and high doses of FE002-SK2 progenitor fibroblasts, respectively.

**Figure 11:**
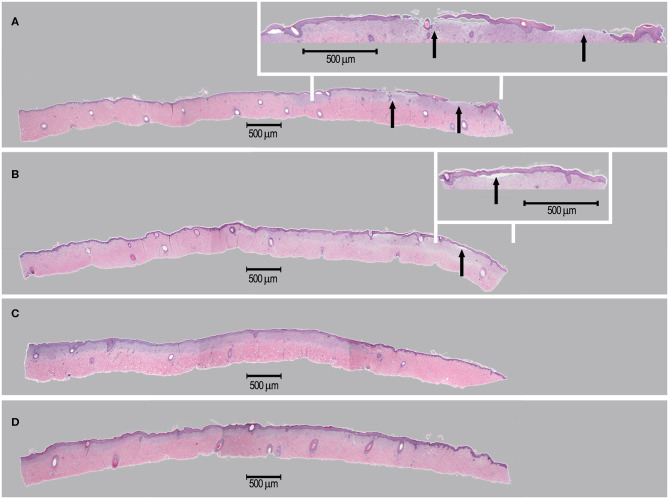
Representative imaging of tissue sections isolated for histological examination. Sections were isolated on Day 14 and stained using HE. Treatment groups [A–D] consisted in the sham control **(A)**, hydrogel scaffold alone **(B)** and hydrogel scaffolds with low **(C)** and high doses **(D)** of FE002-SK2 progenitor fibroblasts, respectively. Arrows in **(A,B)** indicate observable epidermal detachment.

During the study period, administered test articles did not affect body weight of pigs, neither did they cause wound infection, edema, maceration, abnormal inflammation or other adverse effects. Serum TNF-α and IL-1 concentrations were not significantly modified throughout the study period. Serum IgG and IgM concentrations were slightly elevated from Day 0 (IgG 199 ± 105 mg/dL; IgM 119 ± 14 mg/dL) to Day 14 (IgG 343 ± 116 mg/dL; IgM 158 ± 17 mg/dL). The monocyte counts were transiently elevated on Day 3 but returned to baseline levels on Day 14. These immunological changes were interpreted as normal for pigs with trauma and treated with human cells. The wound healing rates were similar for all treatment groups (extensive data not shown). However, on Day 6, though statistically non-significant, healing rates of wounds in group D (scaffold and high dose of progenitor fibroblasts) were found to be higher than those of wounds in group A (sham control). On Day 9, considering wound healing rates (presented in [%] with associated standard deviations), the wounds in group B (scaffold alone) (100.00 ± 0.00), C (scaffold and low dose of fibroblasts) (99.78 ± 0.49), and D (scaffold and high dose of fibroblasts) (100.00 ± 0.00) were nearly fully healed while wounds in group A were not fully healed (95.50 ± 9.09). On Day 14, the scars resulting from wounds in group D were less apparent (Figure 10T). Microscopic observation of HE-stained tissue biopsies showed normal newly healed wounds (Figure 11). Epidermal cells appeared differentiated and stratification was observed in the external layers, while cuboidal cells populated the basal layer. Follicles and dermal papilla were in formation. Interestingly, observation of the tissue sections from all wounds (3 sections *per* wound) revealed that the epithelium appeared to attach better to the dermis in the wounds treated with progenitor cells (Figures 11C,D). Statistical significance was not confirmed because of the small number of test subjects.

## Discussion

Cellular therapies are valuable assets in the endeavors of repairing, restoring or optimizing tissue and organ functions. Efficient combinations thereof with traditional surgical techniques or tissue engineering can demonstrably lead to additive or synergistic beneficial effects on the patients' health momentum (Montjovent et al., 2004; Bach et al., 2006; Loebel and Burdick, 2018; Costa-Almeida et al., 2019). Various considerations are critical during the cell source selection process, related to availability of said therapeutic cell sources, traceable characterization thereof, inherent expansion potential, regenerative potential and applicability to engineered bio-constructs (Doyle and Griffiths, 1998; Monti et al., 2012). Optimization of this selection process, with major emphasis set on safety and consistency, has led to the identification and recognition of allogenic primary progenitor cell types as highly promising and efficient candidates for cell therapies (De Buys Roessingh et al., 2006; Mirmalek-Sani et al., 2006; Grognuz et al., 2016; Kim et al., 2018).

Medical needs in the domains of burns and chronic cutaneous wound management are difficult to meet, due to the complex nature of wound bed environments and the delicate process of coordinated responses governing wound closure. Split-thickness skin autografting remains a gold standard of care for numerous complex cutaneous affections, yet negatively contributes to the burden of patients by implying the creation of a donor site wound. High morbidity is the consequence of large surface wounds and delayed healing, as the opportunity for infection is wider (Simman and Phavixay, 2011). Heavy scarring can in turn cause durable and extensive psychological trauma to be borne by patients. Optimized wound management is therefore critical for individual survival and well-being, particularly in burn victim populations, where primary wounds and donor site wounds are constant concerns. An effective therapeutic solution allowing for regeneration of healthy and functional skin within optimal timeframes would widely benefit both patients and medical staff (De Buys Roessingh et al., 2015; Li and Maitz, 2018). Numerous therapeutic solutions already exist on the market with the indication of favoring wound healing processes. Dressings and topical agents for burns and chronic wounds fulfill different patient- and wound-specific needs. Products typically used in burn centers comprise vaseline gauze (e.g., Jelonet™), adhesive film dressings (e.g., Tegaderm™), hydrocolloids (e.g., DuoDERM®), alginate-based products (e.g., Kaltostat®), soft silicon or foam based products (e.g., Mepitel® Ag or PolyMem®), hydrofibers (e.g., Aquacel®), or cell-based therapy products (e.g., OrCel®, Apligraf®, Dermagraft®, TransCyte®) (Dhivya et al., 2015; Varkey et al., 2015; Borda et al., 2016). Clinical evidence indicates that dermal cells used in a therapeutic product promote optimal and durable tissue repair (Spiekstra et al., 2007; Akita et al., 2008). The CHUV Burn Center uses both cultured epithelial autografts (CEAs) and cultured dermal-epidermal autograft (CDEAs) for specific indications and with positive results (Rheinwald and Green, 1975; Abdel-Sayed et al., 2019). Notwithstanding said results obtained with existing cell-based products and aforementioned therapies, cost-effectiveness, availability delays and product homogeneity between production batches remain of major concern. Optimized quality control and economic rationale are therefore main advantages of the described Swiss progenitor cell technology, which in turn positively benefit each step of product development.

Large and heterogenous arrays of biological materials and cultured progeny cells present interesting therapeutic potential for human medical applications in regenerative medicine. Both animal and human donors can be evaluated, at various ages of physiological development, with inherent problematics for each cell type to potentially be used in a therapy. The focus of applied research in academic and industrial settings comprises embryonic stem cells, umbilical cord cells, fetal cells, adult stem cells, platelets, placenta and amniotic fluid cells, amongst many others (Heathman et al., 2015; Mount et al., 2015). Many of these potential sources require specific handling or manipulation to orient or stabilize the differentiation and self-renewal capabilities of cultured progeny cells, which are to be used for defined therapies. Notwithstanding the diversified offer of donor sources and technological advances, major current restrictions on the developmental path of cell-based products remain related to technical limitations. In most cases, sub-optimal intrinsic parameters of selected cell populations increase the complexity and cost of the pathway to product development and market approval (Heathman et al., 2015; Mount et al., 2015). Such hindering parameters comprise feeble cellular proliferation capacity, plasticity in the maintenance of a differentiated phenotype, possible transmission of communicable diseases or low banking consistency and low *in vitro* stability (Rayment and Williams, 2010; Ratcliffe et al., 2011; Abbasalizadeh and Baharvand, 2013; Heathman et al., 2015; Hunsberger et al., 2015). A given example is the use of allogenic fetal keratinocytes for treating burns and chronic wounds (Zuliani et al., 2013; Tan et al., 2014). Although a combination of allogenic cell populations (fibroblasts and keratinocytes) in a therapeutic product is conceptually interesting, many technical barriers arise and hinder the pragmatic establishment of relatively large and consistent keratinocyte cell banks. This aspect is confirmed by the clinical experience around CEA/CDEAs, whereas keratinocytes are relatively more complex and fastidious to obtain in sufficient numbers. The data presented herein further supports the concept that allogenic keratinocytes are not required, as progenitor fibroblasts would exert potent stimulatory effects on resident keratinocytes to facilitate the healing process.

Cultured primary progenitor dermal fibroblasts from one organ donation such as the FE002-SK2 cell source meet the stringent technical requirements pathing the way to development of allogenic biological therapeutic products (Quintin et al., 2007; Applegate et al., 2009; Larijani et al., 2015). Progenitor cells benefit from extensive historical industrial use, attesting to their safety and stability, whereas many vaccines were produced using such biological substrates since the 1950s (Hayflick et al., 1962; Olshansky and Hayflick, 2017). Such cells were and are isolated by bioprocessing specific fetal tissues, which exist following the embryonic stage of development (i.e., after 9 weeks of gestation). Careful selection and screening of the mother-donor coupled with extensive testing of the progeny cultured cells assures minimal risk of viral, fungal or bacterial disease transmission (Supplementary Figures 1, 2) (Quintin et al., 2007; Applegate et al., 2013). Progenitor cells are differentiated and characterized by high expansion and regeneration potential, while presenting low immunogenic and tumorigenic properties after transplantation (Figure 1) (Doyle and Griffiths, 1998; Quintin et al., 2007). Importantly, these cells do not require growth factor cocktails or feeder-layers for *in vitro* expansion, contrasting with undifferentiated mesenchymal stem cells or primary keratinocytes (Doyle and Griffiths, 1998; Ramelet et al., 2009). The tissue-specific properties of the FE002-SK2 cells and their negligible potential for reverting to a more potent state confer optimal stability and homogeneity to cultured progeny populations under standard conditions (data not shown). Such specificities imply lower technical and financial requirement than embryonic stem cells (ESCs) or induced pluripotent stem cells (iPSCs) for example and widely benefit the industrial product manufacture step. Concretely, end-product benchmarking favors the use of PBBs in the CHUV Burn Center, as previously reported (Abdel-Sayed et al., 2019).

The robustness and advantages of progenitor cell types such as the FE002-SK2 source are therefore mainly based on their isolation process, extensive expansion capacity, minimal growth requirements, excellent biocompatibility with engineered scaffolds and high resistance to oxidative stress. PCBs, MCBs, and WCBs of cultured progenitor cells can be developed rapidly and efficiently while safety testing is continuously performed throughout the bioprocessing and manufacturing workflow (Figure 3) (Quintin et al., 2007). Therapeutic applications require cultured fetal cells to be used up to two-thirds of their documented and validated *in vitro* life-span, as to assure consistency of important biological properties including total protein concentration, gene expression and biological activity (Quintin et al., 2007). Notwithstanding the latter and due to FE002-SK2 progenitor dermal fibroblast source robustness and expansion possibilities, over 39 billion skin bioengineered constructs of 9 × 12 cm can potentially be produced from a dedicated tiered cell bank, which can be cryopreserved for decades. Establishing such cell banks enables the use of consistent and safe starting biological materials, with maximized efficiency and optimized industrial costs, for various applications in tissue engineering and regenerative medicine (Abbasalizadeh et al., 2017; Pigeau et al., 2018). This can assure rapid on-demand availability of consistent end-products once the market is reached, as stored vials of cells at defined passages are directly used for therapeutic product preparation in the clinic. Contrasting with autologous cell therapies, extensive culture periods associated with majored costs and risks of contamination would therefore no longer be required (Quintin et al., 2007; Haack-Sørensen and Kastrup, 2011; Hunt, 2019). Inter-individual variability is also taken out of the equation in an allogenic therapeutic approach, allowing for standardization and optimization of bioprocessing technologies and biologic construct manufacture. Indeed, the consistency, stability and robustness in processing the biological starting material is paramount in assuring optimal safety and therapeutic quality of the end-product to be applied to the patient. An optimal cell source therefore allows for realistic clinical translation, industrial transposition and market implementation within modern regulatory frameworks (Quintin et al., 2007; Larijani et al., 2015; Marks and Gottlieb, 2018).

Up-scaling of FE002-SK2 cell banking, continued translational development and preparation of clinical trials has required and requires many steps. Determination and characterization of key elements including identity, purity, sterility, stability, safety, and efficacy are needed for cellular-based products. Regulations impose strict criteria for the production and manufacturing environment used for cell-based therapies destined for clinical trials and treatments (Ratcliffe et al., 2011; Abbasalizadeh and Baharvand, 2013; Hunsberger et al., 2015). Therefore, working with highly consistent biological starting materials allows for efficiency and safety optimization in the manufacturing process of therapeutic products. In order to allow large numbers of patients to be able to benefit from such therapies, the availability of the biological material needs to be optimized. Therefore, the materials and the *ad hoc* technology need to be easily transposable to different countries under consistent quality specifications (Hunsberger et al., 2015).

Based on extensive in-house experience, implementing technology transfers for industrial-scale banking of allogenic progenitor cells remains tedious to date. Each step of the process needs to be optimized and tested, ensuring that the use by different entities is in accordance with the original banking protocols and legal constraints of the Swiss Fetal Transplantation Program (Applegate et al., 2013). Notwithstanding the technical and administrative barriers, successive and successful technology transfers have been carried out during the last decade with the FE002-SK2 cell source, both in Switzerland and around the globe (Figure 4). The first transposition and industrial up-scaling was performed in Scotland (BioReliance) and enabled the establishment of extensive GMP cell banks (Figures 3, 4). In parallel, clinical batches of FE002-SK2 progenitor fibroblasts are continually manufactured in-house in the GMP-certified Cell Production Center for the CHUV Burn Center (Figure 4). Close inter-disciplinary collaborations have enabled the local transfer of technology and the continuous treatment of severe burn victims in Lausanne. International research and clinical developments based on the FE002-SK2 cell source are led by TWB, which are currently in clinical trials (approved in Japan, Taiwan, USA; ongoing in Japan, Taiwan). Technological transposition from applied research to commercial industrial settings was successfully carried out during the last decade *via* a CHUV spin-off (Elanix Sàrl). The acquired and specific experience in transposition underlined that a very robust cell source and proper oversight were of utmost importance in ensuring optimal, rational, safe, and efficient use of cell starting materials. Consistency of the cell banks must be met by consistency in the use made of the materials. Achieving multi-center transposition then allows to work with different regulatory frameworks, which stimulates and directs the applied and clinical research in complementary ways.

In the Lausanne University Hospital, progenitor cells were used in clinical settings particularly for severe burns and acute or chronic wounds in human patients, generating over two decades of pioneer clinical experience in pediatric and geriatric populations (Hohlfeld et al., 2005; Ramelet et al., 2009; De Buys Roessingh et al., 2015). The Progenitor Biological Bandages used in the clinic to this day are engineered wound coverage constructs composed of an equine collagen scaffold (9 × 12 cm) carrying clinical-grade FE002-SK2 progenitor fibroblasts (Abdel-Sayed et al., 2016, 2019). On-demand availability to clinicians allows for application of such constructs in the early phases of treatment of trauma victims. The PBBs are used to favor healing of second degree superficial and deep burns, as well as donor site grafts routinely used in the Burn Center. The overall therapeutic goal is to limit the damage to the burned tissues and restrict the need for donor site skin-grafting. This goal was already reached with the treatment of numerous pediatric burn victims to date (Hohlfeld et al., 2005; De Buys Roessingh et al., 2006). The implementation of the Bandages to broader routine clinical use is under examination and will be the object of a new internal clinical trial (Priority Project Bru_PBB). Retrospective studies have already yielded preliminary safety results for the therapy on adult and pediatric patients (data to be published). Standardized comparison with standards of care for burn patients must be performed, in order to assess the positive impacts and therapeutic benefits of the treatment. The Progenitor Biological Bandages developed in the CHUV were also used as a base in the Project Platform *SwissTransMed*, associating antimicrobial factors for the treatment of burn patients (Abdel-Sayed et al., 2016).

Most recent applied research in Switzerland and Taiwan using the FE002-SK2 cell source has aimed toward elucidation of putative mechanisms of action which intervene and possibly mediate therapeutic effects after clinical delivery. Progenitor fibroblast preparations and derivatives thereof (cell extracts, replication-inactivated cells and conditioned media) were found to have demonstrable high bioactivity in defined *in vitro* assays for primary keratinocyte migration and proliferation stimulation. In particular, (a) FE002-SK2 cells facilitated primary keratinocyte migration in Transwell® assays (2.7-fold mean relative increase in migrated population counts, Figure 5, Table 1), (b) FE002-SK2 conditioned media facilitated *in vitro* epithelial gap-filling (significant decrease in filling time, Figure 6), (c) FE002-SK2 cell extracts promoted keratinocyte proliferation (strong increase in total cell counts after 7 days of stimulation, Figure 7, Table 2) and (d) FE002-SK2 cells and growth-arrested variants promoted keratinocyte proliferation (strong feeder-layer effect, Figures 8, 9, Table 3). Hence, quantified *in vitro* evidence of biological effects has been documented for the cell type of interest and is crucial for product characterization and standardization, addressing both manufacturing quality control and regulatory requirements (Hunsberger et al., 2015; Marks and Gottlieb, 2018). By extension, such results support the use of pure fibroblastic populations as allogenic therapeutic agents, without conjugation to technically demanding keratinocytes, due to the potent stimulatory effects described hereabove.

Further analysis of the biomarkers found in co-culture conditioned media may help to elucidate the mechanisms underlying the therapeutic stimulation of wounded tissues. Beneficial effects are indeed suspected to be of trophic nature, relying on patient cell stimulation by the applied engineered constructs and biological materials, rather than engraftment of allogenic cells (Spiekstra et al., 2007; Werner et al., 2007; Barrientos et al., 2008). In addition, liberation of matrix proteins by allogenic therapeutic cells probably supports and promotes structural modifications occurring during tissue remodeling. Biochemical paracrine signaling, through the release of growth factors and cytokines in well-proportioned combinations, probably acts by favoring a return to cutaneous homeostasis after dynamic and multi-step healing of the skin (Wojtowicz et al., 2014). The results presented in Table 4 suggest clear synergistic effects of cell-cell interactions relative to the expression of biomarkers of interest. These biomarker quantifications allow for hypotheses establishment around the role of the biological therapeutic material in the wound environment, keeping in mind the vast differences between the *in vitro* assay settings and a patient's damaged tissue. Hypotheses are formulated hereunder based on the relative quantities of biomarkers found in the co-culture conditioned media.

PAI-1 (Plasminogen activator inhibitor-1) is a serine protease inhibitor which inhibits degradation of fibrin by plasmin, as well as the activity of matrix metalloproteinases (MMPs). During wound healing, elevated levels of PAI-1 inhibit uPA/tPA/plasmin and plasmin-dependent MMP activities, which may help to facilitate wound healing, by modulation of the intrinsic local repair mechanisms (Providence et al., 2008). Relatively high levels of PAI-1 in the FE002-SK2 cultures (13,922 pg/mL) and co-cultures (19,550 pg/mL), coupled with an apparent low inter-stimulation of its expression in the co-cultures (resulting additive contributions of both cell populations) indicate that the progenitor fibroblasts are responsible for allogenic contributions of PAI-1 (Table 4). TIMP1 and TIMP2 are tissue inhibitors of metalloproteinases, which also inhibit the degradation of tissue matrix. In chronic wounds, the ratio of TIMP/MMP may be too low, which delays wound healing (Krishnaswamy et al., 2017). Co-culture settings had a negligible or slightly antagonistic effect on the production of TIMPs by the cell populations, but it appears again that the progenitor fibroblasts are responsible for the presence of relatively higher levels of these biomarkers in the conditioned media (20-40X higher TIMP levels in fibroblast cultures *vs*. keratinocyte cultures, Table 4). Galectin-7 (Gal-7) binds to beta-galactosids and is mainly expressed in stratified epithelial cells, including keratinocytes. Gal-7 is involved in cell-cell and cell-matrix contacts. Gal-7 gene (*LGALS7*) knockdown results in reduced differentiation and increased proliferation of keratinocytes (Chen et al., 2016). This is coherent with the increased keratinocyte proliferation in the co-cultures in this study, whereas Gal-7 levels were decreased. Several additional factors (e.g., AR, CXCL1, CXCL5, CXCL8, CXCL16, Follistatin, IGFBP2, IL-6, IL-6R, MCP-1, and RANTES) were found to be highly expressed in the co-culture conditioned media, while very low levels were detected in the media of single population cell cultures. These factors may be produced by keratinocytes or FE002-SK2 fibroblasts under the conditions of keratinocyte-fibroblast interactions and may play a role in facilitated keratinocyte migration and proliferation *in vitro*. *In vivo* effects can be hypothesized based on the biomarker data but would need more extensive investigation on clinical patient samples. CXCL5 (ENA-78, epithelial-derived neutrophil-activating peptide 78) is produced following stimulation of cells by the inflammatory cytokines IL-1 or TNF-alpha. ENA-78 stimulates the chemotaxis of neutrophils *via* CXCR2, possessing angiogenic properties and stimulating proliferation and migration of keratinocytes (Zaja-Milatovic and Richmond, 2008; Kroeze et al., 2012). The highly synergistic effect of co-cultures on the ENA-78 levels and the naturally highly inflammatory nature of deep skin wounds are coherent with the hypothesis that allogenic fibroblasts potently interact with host keratinocytes in view of repair and regeneration stimulation. CXCL1 [Gro, Gro-alpha, neutrophil-activating protein 3, or chemokine (C-X-C motif) ligand 1], which elicits its effects through the chemokine receptor CXCR2, activates EGFR and stimulates proliferation of epithelial cells. CXCL16 acts as a mediator of innate immunity by attracting CXCR6-expressing cells, such as activated T cells and natural killer T (NKT) cells. CXCL16 is constitutively expressed on the surface of human epidermal keratinocytes, released upon cell activation or photodamage (Scholz et al., 2007).

IL-6 is known to regulate a broad spectrum of immune responses, to stimulate the proliferation of many types of cells and as being vital to wound healing (Gallucci et al., 2000). CXCL8 (IL-8) is a chemokine produced by macrophages and other cell types such as epithelial cells, airway smooth muscle cells and endothelial cells, eliciting its function through CXCR1 and CXCR2. IL-8 is known to recruit neutrophils and to be a potent promoter of angiogenesis, while stimulating proliferation and migration of cultured keratinocytes (Jiang et al., 2012; Sobel et al., 2015). Follistatin (activin-binding protein) has a modest angiogenic effect on endothelial cells and is strongly synergistic with bFGF (basic fibroblast growth factor). Binding of follistatin to activin inhibits the mitogenic function of activin. Follistatin is considered a growth regulator of epithelial cells (Antsiferova et al., 2009). Therefore, synergistic effects of co-cultures on IL-8 and follistatin levels are additional indicators of the trophic dialogues that may take place between allogenic fibroblasts and patient resident keratinocytes in view of therapeutic immune modulation and repair stimulation. The synergistic effects of the co-cultures on the levels of CXCL1, 5, 8, 16, and IL-6 support the hypothesis that under the influence of the progenitor fibroblasts, the patients innate immunity may be stimulated to combat dangerous exogenous pathogens, which is then followed by cell migration and proliferation for regenerating tissues.

For cell-based therapeutic product development submission files, regulatory agencies expect a potency assay pertinent to the clinical indication to be established with the completion of phase II clinical trials, to thoroughly support both proof-of-concept and safety of the product. The quantity of research allocated to biomarker analysis herein was dictated by regulatory requirements, for which stimulation of proliferation and migration coupled to preliminary biomarker analysis was sufficient (for the countries where applications were positively evaluated, i.e., USA, Taiwan, Japan). In developing a cell therapy product, one of the major challenges throughout various product developmental stages (initial research, design of dosage form, development of production process and quality control (QC) method, preclinical toxicology studies, phase I, II, III clinical trials and scale-up manufacturing for marketing) is determining the critical quality attributes (CQAs) or mechanism of action (MOA), which might not be very clear or specific. The keratinocyte migration assay described herein has become part of the actual product release testing. Enhancement of epithelialization rate was targeted as a specific mechanism of action within the indication of treating burns and split thickness wounds, hence the investigation of the ability of fetal progenitor fibroblasts to promote keratinocyte proliferation and migration. Subsequent detailed analysis of culture system protein markers was undertaken in view of further understanding the possible attributing factors of the observed stimulatory effects. However, wounded environments drastically differ from controlled culture conditions of *in vitro* models, whereas results yielded by the biomarker study therefore remain speculative, as markers characterized *in vitro* may or may not affect endogenous keratinocyte behavior on patient wounds. Within the specific context of product development and the relative scarcity of resources and workpower, more advanced biomarker investigations were not yet considered. Indeed, while extremely interesting at a fundamental level, the biomarker investigations were more important for implementing QC assay specifications within the product development process and approval thereof. The actual product QC is performed by quantification of specific biomarkers to assess cell batch quality and to standardize therapeutic protocols and delivered doses. Similarly, the *in vivo* preclinical preliminary safety and efficacy investigations were not performed within robust or statistically significative settings, as the minimal regulatory requirements were followed, in order to rapidly move along to human clinical trials. The fundamental differences between academic research and industrial-driven development are underlined by this specific issue, as resource constraints and respective objectives differ, yet reconciliation of both aspects is necessary for coherence of the overall message and setting of perspective for considerations about translational therapeutic product development.

Overall, the summarized and probable benefits of progenitor fibroblast therapeutic applications for wound healing induction reside in the secretion of cytokines, growth factors and other ECM proteins onto damaged tissues. In the inflammatory context of cutaneous wounds, cellular proliferation and migration ultimately lead to tissue remodeling (Werner et al., 2007; Barrientos et al., 2008; Providence et al., 2008). Therapeutic stimulation of these physiological processes, which may be complemented by immune-modulation and angiogenesis, can reasonably be conceived after application of progenitor dermal fibroblasts. Data from the *in vivo* porcine study confirmed safety of application of the biological constructs on animal subjects. Low immunogenicity of the products in an inter-species setting strengthened the rationale for specific human biological starting material selection and processing, using immune-privileged sources. Comparative observations between the test groups yielded preliminary indication of superior epidermal attachment at the time of biopsy harvest for the wounds treated with cellular products (Figures 10, 11). While healing rate enhancements were subjectively noted with the use of therapeutic progenitor cells, larger sample groups and specific clinical endpoints shall be further adopted for stringent evaluation of product clinical efficacy. In the specific context of product development, these data will in fact be obtained in the early clinical trial phases (ongoing). Notwithstanding the latter, such *in vivo* evidence complements the extensive clinical experience acquired over the years around the use of PBBs on pediatric and adult human patients suffering from acute and chronic wounds (Hohlfeld et al., 2005; De Buys Roessingh et al., 2015; Abdel-Sayed et al., 2016, 2019). Human safety and comparative efficacy studies are on-going in Asia and pending in the CHUV, in order to assess and quantify the therapeutic gains to be obtained with FE002-SK2-based biologic constructs for wound management.

As cell therapies, the final products yielding FE002-SK2 progenitor fibroblasts must meet numerous standards before being allowed to reach the market. Considerable and continuous efforts are needed to meet the regulatory requirements of respective countries. In parallel, further research needs to be conducted, in order to optimize the logistics and efficiency of the proposed clinical protocols. Indeed, the state-of-the-art protocols detail the use of viable cells preserved in ultra-low temperature freezers or cryogenic storage. A major technical and logistical advantage would be gained by further processing the cellular starting material, in a manner which would respect and maintain biological characteristics, to obtain off-the-shelf stabilized products to be stored at 4°C (Ratcliffe et al., 2011; Abbasalizadeh and Baharvand, 2013; Hunsberger et al., 2015). Further advances in drug delivery options for progenitor fibroblasts will synergistically benefit the regeneration of damaged tissues. Nonetheless, current practices using allogenic progenitor cell-sources enable drastic reduction of clinical availability delays while maximizing consistency and stability of starting biological materials. Indeed, one single defined organ donation in 2009 enabled the establishment of extremely robust and extensive Parental, Master and Working Cell Banks with the FE002-SK2 cell source. Traceability and safety are paramount for Investigational Medicinal Product Dossiers and Investigators' Brochures preparation in view of clinical trials using cellular products (Rayment and Williams, 2010; Heathman et al., 2015). An optimal therapeutic cell source to meet such stringent requirements set forth in this translational work was therefore devised under the Swiss Fetal Transplantation Program. The FE002-SK2 source has supplied research and clinical applications for the last decade and will continue to do so globally. Vast preliminary experience attests to the strong potential of the progenitor technology to be used as a therapeutic product. The ongoing steps to implement FE002-SK2 product use in clinical practice are crucial and well underway, determining the impact and benefits to be gained by patient populations in general.

## Conclusion

Within the well-defined and regulated context of cell therapy product development, the advantages of a safe and consistent biological starting material have been presented herein using the example of progenitor fibroblasts. The large quantity of data which has been generated to date around the FE002-SK2 progenitor cell source is consistently stressing the importance of optimization and standardization in manufacturing processes. The first-hand experience around industrial transposition of pioneer biomedical technology resumed herein attests to the complexity in reaching technical success, a prerequisite for sound product development. Pragmatically harnessing the inherent variability characterizing biologic substrates allows for better implementation of their high therapeutic potentials, both in the regulatory and clinical settings. Technical aspects regarding cell source optimization and whole-cell bioprocessing are not only scientifically relevant but lay the foundations of efficient and widely-available cell-based therapies. A single organ donation was sufficient for the last decade of applied research and clinical investigations. The implementation of optimized bioprocessing methodology and well-devised industrial-scale GMP biobanking of the FE002-SK2 cell source have demonstrated that extensive and consistent clinical-grade cell banks can be established. Although autologous cell therapies remain of high interest and are preferred regulatory-wise, bringing more evidence to the benefits of consistent allogenic products will surely and naturally direct toward a paradigm shift in the near future. The unique clinical experience established in Switzerland combined with the ongoing clinical trials in Asia will surely confirm the therapeutic benefits of the unique FE002-SK2 cell source. Through further efforts directed at clinical translation and commercial implementation, quality and efficiency of therapeutic care will be optimized. Musculoskeletal tissue affections and overall health of patients worldwide will therefore surely benefit from the unique progenitor cell technology developed in Switzerland.

## Data Availability Statement

All datasets generated for this study are included in the article/Supplementary Material.

## Ethics Statement

FE002-SK2 primary progenitor cells were isolated from the FE002 organ donation according to a validated protocol, approved by the State Ethics Committee (University Hospital of Lausanne—CHUV, Ethics Committee Protocol #62/07: Development of fetal cell banks for tissue engineering, August 2007). The FE002 donation was registered under the Federal Transplantation Program and its Biobank, complying with the laws and regulations within both programs. Obtention and use of progeny cells follow regulations of the Biobank of the Department of Musculoskeletal Medicine in the CHUV. Cell bank safety testing assays performed by BioReliance which involved animal subjects were performed in accordance with the OECD Principles of Good Laboratory Practice (GLP) and individual studies were conducted after proper internal ethical considerations and validation. The porcine study in Taiwan was carried out at the Agricultural Technology Research Institute under GLP standards after proper internal ethical considerations and validation.

## Author Contributions

AL, PL, NH-B, B-RS, and LA: Study conception and design. AL, PL, CS, MM, AB, NH-B, WR, B-RS, and LA: Acquisition of data. AL, PL, NH-B, B-RS, and LA: Analysis and interpretation of data. AL, PL, and B-RS: Drafting of the manuscript. AB, WR, NH-B, B-RS, and LA: Critical revision. AL, PL, CS, NH-B, MM, AB, WR, B-RS, and LA: Acceptance of final manuscript.

## Conflict of Interest

CS, NH-B, AB, WR, and LA were the Founders of Elanix Sàrl, which was constituted for the industrial transposition of the bioprocessing technology out from the CHUV University Hospital. LA is listed as Inventor on the patent describing the bioprocessing technology for progenitor cell banking and is currently the *ad-interim* Administrator of Elanix Sàrl. PL and B-RS are employees of Transwell Biotech Co. Ltd. in Taiwan and hold shares in said Company. AL was employed by company Tec-Pharma SA. The remaining author declares that the research was conducted in the absence of any commercial or financial relationships that could be construed as a potential conflict of interest.

## Abbreviations

ATMP: Advanced Therapy Medicinal Product
ATRI: Agricultural Technology Research Institute
BSA: Bovine Serum Albumin
CDEA: Cultured Dermal-Epidermal Autograft
CEA: Cultured Epithelial Autograft
cGMP: current Good Manufacturing Practices
CHUV: Centre Hospitalier Universitaire Vaudois
CMV: Cytomegalovirus
CPC: Cell Production Center
CQA: Critical Quality Attribute
DAPI: 4′,6-diamidino-2-phenylindole
DK-SFM: Defined Keratinocyte-Serum Free Medium
DMEM: Dulbecco's Modified Eagle Medium
DMSO: Dimethyl Sulfoxide
DPBS: Dulbecco's Phosphate-Buffered Saline
EBV: Epstein-Barr Virus
ECACC: European Collection of Authenticated Cell Cultures
ECM: Extra-Cellular Matrix
EDTA: Ethylenediaminetetraacetic acid
EOP: End Of Production
EOPCB: End Of Production Cell Bank
FBS: Fetal Bovine Serum
FDA: Food and Drug Administration
GLP: Good Laboratory Practices
GMP: Good Manufacturing Practices
HbsAg: Hepatitis B virus surface antigen
HBV: Hepatitis B Virus
hCMV: Human Cytomegalovirus
HCV: Hepatitis C Virus
HE: Hematoxylin and Eosin
HepB/C: Hepatitis B and C
HHV-6/7/8: Human Herpes Viruses types 6, 7 and 8
HIV-1/2: Human Immunodeficiency Viruses types 1 and 2
HTLV-1/2: Human T-cell Leukemia Viruses types 1 and 2
HUG: Hôpitaux Universitaires de Genève
IgG: Immunoglobulin G
IgM: Immunoglobulin M
ITRI: Industrial Technology Research Institute
K-SFM: Keratinocyte-Serum Free Medium
LOD: Limit Of Detection
MCB: Master Cell Bank
MMC: Mitomycin C
MOA: Mechanism Of Action
NIH: National Institutes of Health
PBB: Progenitor Biological Bandage
PBS: Phosphate-Buffered Saline
PCB: Parental Cell Bank
PMDA: Pharmaceuticals and Medical Devices Agency
Px: Passage number x
QC: Quality Control
Q-PCR: Quantitative Polymerase Chain Reaction
RNA: Ribonucleic Acid
SV40: Simian Virus 40
TFDA: Taiwan Food and Drug Administration
UTR: Regenerative Therapy Unit
WCB: Working Cell Bank.
