# Supplementary material for: Bringing Safe and Standardized Cell Therapies to Industrialized Processing for Burns and Wounds
Source: Front Bioeng Biotechnol. 2020 Jun 19;8:581. doi: 10.3389/fbioe.2020.00581 (PMC7317026; doi:10.3389/fbioe.2020.00581)
Supplement: Supplementary file 1 [file Image_1.pdf]

SUPPLEMENTARY MATERIAL

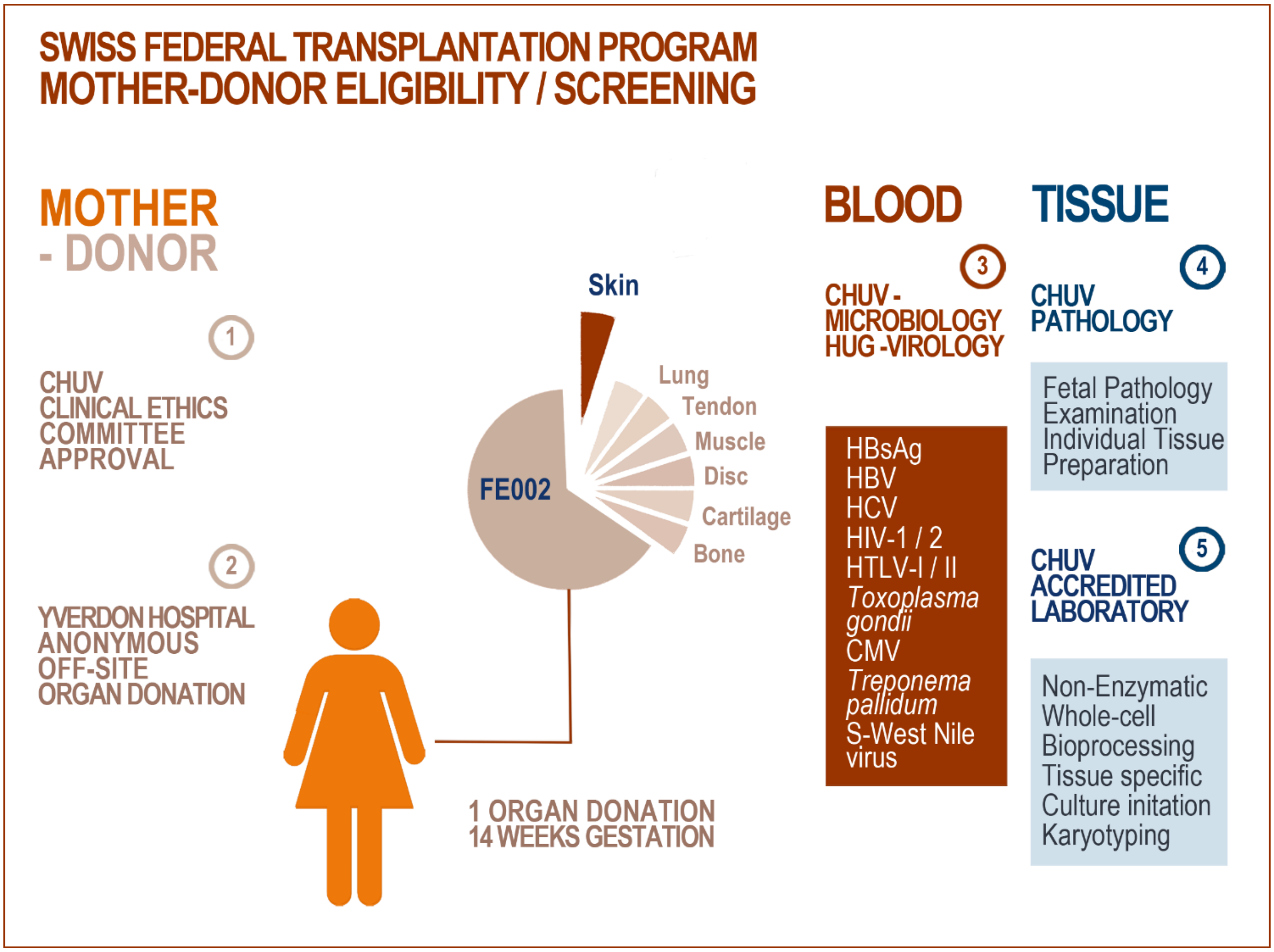

Supplementary Figure 1 | Following the devised workflows of the Swiss Fetal Transplantation Program, the protocols for Fetal Biobanking were approved by the CHUV University Hospital Medical Ethics Committee (Protocol #62/07: "Development of fetal cell banks for tissue engineering", August 2007). **(1)** Written and oral consent were obtained for the Transplantation Program, associated to an appropriate period for reconsideration. **(2)** The FE002 donation (14-week-old male fetus of 14 cm and 44.4 g) took place at the Gynecology and Obstetrics Service of the Hospital of Yverdon-les-Bains and was immediately transported to the CHUV in Lausanne. **(3)** Rigorous screening of the mother donor through anonymous health questionnaires and extensive bloodwork (repeated after 3 months) excluding sero-conversion for the target pathogens were realized by the medical team, the CHUV Institute of Microbiology in Lausanne and the Virology Laboratory in the University Hospital of Geneva (HUG) under appropriate accreditations. Test results were negative and/or within specifications. **(4)** For confirmation of anatomical normality and acquisition of specific tissue fragments for bioprocessing, the FE002 donation was procured to the CHUV Institute of Pathology, where a fetal pathologist confirmed that the donation was a 14-week-old male fetus of Caucasian ethnicity. No major abnormality was observed for the umbilical cord, cardiovascular, respiratory, urogenital, digestive, endocrine, hematopoietic, musculoskeletal or central-nervous systems. Several distinct tissues were individually acquired and separately conditioned during micro-dissection, including ventral and dorsal skin, the latter subsequently becoming the material source for establishment of the FE002-SK2 cell type. **(5)** FE002 skin samples were then transferred to an accredited bioprocessing laboratory for non-enzymatic cell isolation and *in vitro* culture initiation.

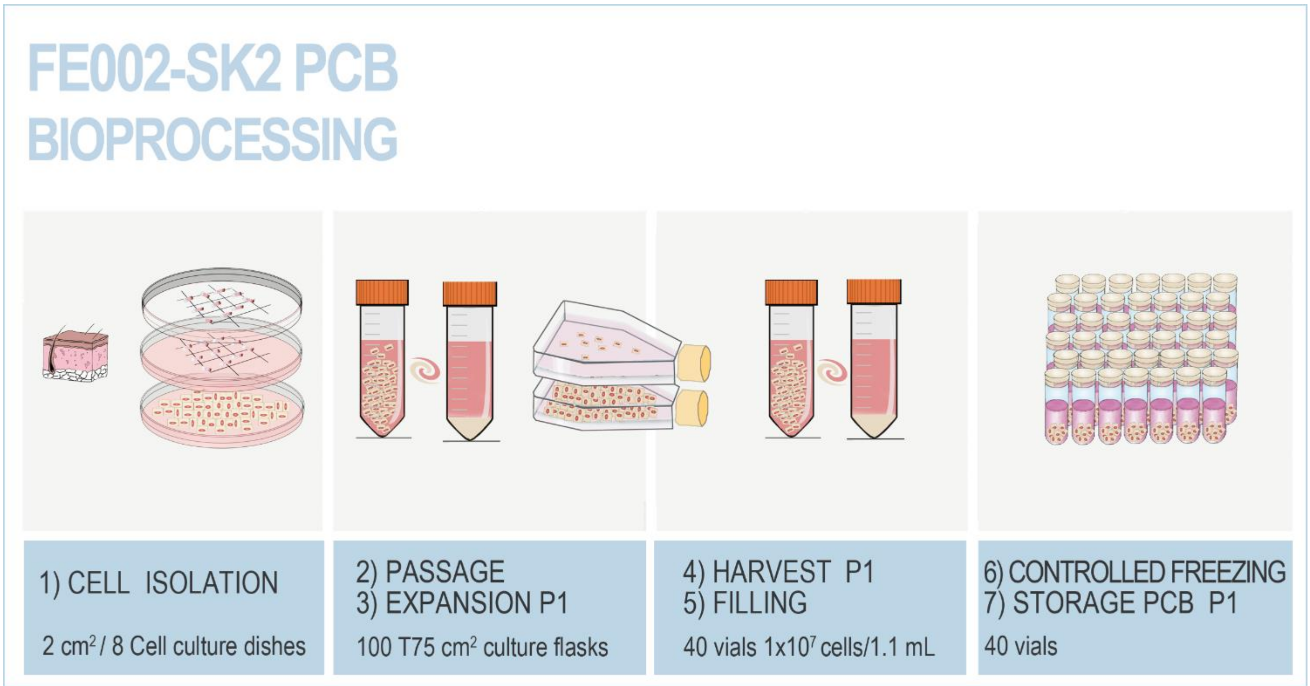

Supplementary Figure 2 | Overview of non-enzymatic whole-cell bioprocessing of FE002 skin samples. **(1)** Dorsal skin fragments were made available by the pathologist in phosphate-buffered saline (PBS CHUV 1X; NaCl 6.8 g/L, Na_2HPO_4 1.5 g/L, KH_2PO_4 0.4 g/L, N°100 0 324, Laboratorium Dr. G. Bichsel AG, Switzerland). The tissues were rapidly transferred (4°C conservation) to a dedicated and accredited CHUV facility for bioprocessing. Approximately 2 cm^2 in total usable skin tissue was made available from the FE002 donation. The fragments were washed 3 times for 15 minutes in PBS containing 1% penicillin-streptomycin (N°15140-122, Gibco®, UK). To isolate and initiate adherent cell culture of dermal progenitor fibroblasts, independent tissue sections were then dissected into < 0.5 mm^3 fragments and placed in 10 cm diameter cell culture Petri dishes (N°353003, Falcon®, USA). Tissue culture dishes had been sterilely and deeply scored in a checkerboard pattern using a scalpel. Isolated tissue fragments were placed and attached along the scored plastic regions by gentle mincing. From the original 2 cm^2 tissue biopsy, 8 Petri dishes were planted with homogeneously distributed whole tissue fragments (~5-10 fragments/dish). A small volume of liquid growth medium was dispensed around each fragment to avoid flotation during the first 24-hour period and the dishes were incubated in a 37°C humidified atmosphere with 5% CO_2. The initial growth medium was composed of Dulbecco's Modified Eagle Medium containing 25 mM dextrose, 1 mM sodium pyruvate and L-glutamine (DMEM 1X, N°41966-029, Gibco®, USA) supplemented with 10% clinical-grade fetal bovine serum (FBS, N°10101145, Invitrogen™, USA). Following the first 24 hours of culture, 8 additional mL of growth medium were carefully dispensed in each dish, before the vessels were incubated again. The growth medium was exempt of antibiotic supplementation and was renewed every 2 days. The first outgrowth emitting from the tissue fragments was observed as early as 24 hours after culture initiation. Towards the end of the initial culture period, abundant fibroblasts had migrated outwards from the tissue fragments. Cell outgrowth was allowed to attain 90% confluency before harvest (Passage 0). **(2)** Once optimal banking confluency (90%) was attained for these primary cultures (6 days), each dish was rinsed twice with PBS and submitted to trypsinization (trypsin-EDTA: 0.05% trypsin-ethylenediaminetetraacetic acid, N°25300-054, Gibco®, USA). After complete cellular detachment, the cell suspensions were diluted with equal volumes of initial growth medium for trypsin inactivation and cells were counted using a hemocytometer. The pooled suspensions were then centrifuged at 230 x g for 15 minutes. **(3)** The cells were then resuspended in fresh, sterile and warmed (37°C) Complete Medium and distributed into 100 T75 sterile cell culture flasks (Nunc®, N°153732, USA) to allow expansion of Passage 1 cells. Complete Medium was composed of DMEM, FBS and 200-times concentrated L-glutamine (N°25030-024, Gibco®, USA) in 100:10:1 volumetric proportion. Final L-glutamine concentration was 5.97 mM and the Complete Medium was exempt of antibiotic supplementation. The seeded culture vessels were incubated at 37°C with 5% CO_2. The Complete Medium (15 mL/flask) was renewed every two days until the confluency attained 100%. **(4)** Cells were then harvested by trypsinization and counted, as described hereabove. The cell suspension was then centrifuged for 15 minutes at 230 x g. **(5)** After supernatant removal, the cell pellet was resuspended in a cryopreservation solution composed of DMEM, FBS and dimethyl sulfoxide (DMSO, N°D-2438, Sigma-Aldrich®, USA) in 67.5%; 27.5%; 5.0% proportions at a final viable cellular density of 10^7 cells/1.1 mL. **(6)** Cells (Passage 1) were then frozen in individual aliquots (10^7 cells/1.1 mL, 40 labelled cryovials, N°366656, Nunc®, USA) using Nalgene® Mr. Frosty® Cryo 1°C Freezing Containers (Nalgene®, UK) which were rapidly placed in a -80°C freezer to achieve a -1°C/minute rate of cooling and a corresponding freezing curve. **(7)** After 24 hours in the freezing containers, the cryovials were transferred to liquid nitrogen vapor phase (−165°C) for long term storage. The vials were subsequently stored in separate level-alarm fitted containers in several locations to mitigate risks. This cryopreserved material was defined as the FE002-SK2 Parental Cell Bank (PCB, Passage 1).
